# Inherited long telomeres induce a genome-wide transcriptional response in budding yeast

**DOI:** 10.1093/genetics/iyag174

**Published:** 2026-07-03

**Authors:** Vasilisa Sidarava, David Lydall

**Affiliations:** Biosciences Institute, The Medical School, Newcastle University, Framlington Place, Newcastle upon Tyne, Tyne and Wear NE2 4HH, United Kingdom; Biosciences Institute, The Medical School, Newcastle University, Framlington Place, Newcastle upon Tyne, Tyne and Wear NE2 4HH, United Kingdom

**Keywords:** telomere length, transcriptional response, *Saccharomyces cerevisiae*

## Abstract

Eukaryotes typically maintain telomere length within a defined range. While short telomeres are known to activate DNA damage responses and limit cell proliferation, long telomeres are associated with extended proliferative capacity. The broader cellular consequences of long telomeres are comparatively less well understood. In budding yeast *Saccharomyces cerevisiae*, long telomeres have been shown to influence gene expression at specific loci, but whether long telomeres affect transcription genome-wide has not been reported. Here, we analyzed transcriptomes in a lineage that inherited long telomeres (originally due to a *rif2*Δ mutation). Transcriptomes were assessed over 2 successive generations, including mitotic and meiotic divisions, in the absence of the *rif2*Δ mutation. We show that strains with long telomeres exhibit a distinct gene expression profile, including upregulation of membrane transporters and downregulation of a smaller subset of genes. Both up- and down-regulated genes were distributed across the genome, arguing against a purely telomere proximal effect on gene expression. Affected genes were enriched for Rap1 binding sites, consistent with a model in which long telomeres sequester telomere-associated transcriptional regulators, such as Rap1, and thereby affect gene expression at non-telomeric binding sites for these regulators. Accordingly, the magnitude of transcriptional changes was greatest in strains with the longest telomeres. Together, our findings demonstrate that long telomeres induce a genome-wide transcriptional response that can accompany inherited long telomeres across generations. Similar effects of long telomeres are likely to occur in other eukaryotes, including humans, where long telomeres are associated with disease.

## Introduction

Telomeres are specialized nucleoprotein structures that cap the ends of linear chromosomes and protect the DNA from inappropriate repair, recombination, and degradation (Celli et al. 2006; Wu et al. 2006). Telomere length is typically maintained within an optimal range, and deviations from this range can have significant biological consequences (Armanios 2022).

In humans, abnormal telomere length is associated with distinct pathological syndromes. Short telomere syndromes are associated with impaired stem cell renewal and premature tissue degeneration, leading to manifestations such as bone marrow failure, pulmonary fibrosis, and liver disease (Armanios and Blackburn 2012). Conversely, long telomeres are associated with extended cellular proliferative potential and an increased risk of malignant transformation, likely by permitting additional rounds of cell division and thereby increasing the accumulation of mutations (McNally et al. 2019). At the cellular level, critically short telomeres activate DNA damage signaling pathways that can induce cell cycle arrest, senescence, or apoptosis (d'Adda di Fagagna et al. 2003). However, the effects of long telomeres beyond replicative lifespan remain unclear.

One challenge posed by excessively long telomeres is DNA replication, as telomeric DNA is intrinsically difficult to replicate due to its repetitive, G-rich sequence, and associated protein complexes (Olson and Wuttke 2024). Consistent with this, long telomeres are unstable and can undergo recombination-based trimming events in yeast (Li and Lustig 1996), plants (Watson and Shippen 2007), and human cells (Pickett et al. 2009). Nevertheless, studies in budding yeast have not detected substantial defects in growth, lifespan, or genotoxic stress sensitivity in cells with long telomeres (Harari et al. 2017), suggesting that any detrimental effects are minimal.

In budding yeast, long telomeres can influence gene expression through at least 2 mechanisms. One is the telomere position effect (TPE), whereby telomere-associated silencing factors such as Rap1 and the Sir proteins promote transcriptional repression of genes located near chromosome ends through the spreading of silent chromatin (Gottschling et al. 1990; Brothers and Rine 2022). The other is by competing for DNA-binding regulatory proteins (Buck and Shore 1995; Marcand et al. 1996).

The effects of long telomeres on TPE can be complex. For example, in yeast that carried a single elongated telomere, the long telomere was associated with enhanced silencing of adjacent reporter genes (Kyrion et al. 1993). In contrast, *rif1*Δ mutants, with mostly elongated telomeres, showed reduced silencing at specific subtelomeric reporter genes (Juarez-Reyes et al. 2023), suggesting that the effect of telomere elongation on TPE depends on the context.

Rap1 and the Sir2/3/4 complex also regulate transcription at internal genomic sites (Rusche et al. 2003; Azad and Tomar 2016). Consistent with this, long telomeres enhanced silencing at telomeres and weakened silencing at internal Sir- and Rap1-dependent loci (Buck and Shore 1995). Whether long telomeres induce broader transcriptional changes has not been reported. Here, we address this question by performing transcriptomic analysis of yeast strains with long telomeres, generated through mutation or inherited in the absence of the mutation that stimulated telomere elongation. Our analyses reveal a distinct genome-wide transcriptional response correlating with the inheritance of long telomeres in yeast.

## Materials and methods

### Yeast strains

Strains used in this study are listed in Table 1.

**Table 1.** Strains used in this study.

Strain	Background	Genotype	Origin	Designation
DLY 3001	W303	*MATα ade2-1 trp1-1 can1-100 leu2-3,112 his3-11,15 ura3 GAL + psi + ssd1-d2 RAD5*	Lydall lab collection	*WT*
DLY 8460	W303	*MATa ade2-1 trp1-1 can1-100 leu2-3,112 his3-11,15 ura3 GAL + psi + ssd1-d2 RAD5*	Lydall lab collection	*WT*
DLY 12558	W303	*DLY 8460 rif2*Δ*::KANMX*	(Sidarava et al. 2025)	*rif2*Δ
DLY 12568	W303	*DLY 3001 long VIR telomere*	(Sidarava et al. 2025)	*WT_long1*
DLY 12581	W303	*DLY 3001 long VIR telomere*	(Sidarava et al. 2025)	*WT_long2*

### Yeast propagation and genetic manipulation

Yeast were routinely cultured at 30 °C on YEPD plates (1% yeast extract, 2% peptone, 2% agar, 2% dextrose) supplemented with adenine (75 mg/l). To produce diploids, haploid strains of opposite mating types were mixed on YEPD plates and mated. Zygotes were picked after 3 h at 30 °C and isolated using a tetrad dissection microscope. Colonies derived from individual zygotes were grown on YEPD plates for 3.5 d and subsequently inoculated into 4 ml liquid YEPD and grown overnight at 30 °C. Each culture was then split into 2 equal aliquots: one used for genomic DNA extraction and telomere length analysis, and the other for RNA extraction and sequencing.

### Telomere length detection

Genomic DNA extraction and telomere length analysis were performed as previously described (Sidarava et al. 2025).

### RNA sequencing and downstream analyses

Total RNA was extracted using RNeasy Mini Kit (Qiagen) according to the manufacturer's instructions. RNA sequencing was performed by Novogene Europe. Strand-specific libraries were generated from poly(A)-selected mRNA using the New England Biolabs NEBNext Ultra Directional RNA Library Prep Kit. After fragmentation and cDNA synthesis (with dUTP incorporation for strand specificity), libraries were adapter-ligated, size-selected, and treated with USER enzyme (NEB). This was followed by PCR amplification. Final library quality was assessed using a Bioanalyzer. Sequencing was carried out on the Illumina platform, yielding paired end reads of 150 bp in length, with approximately 20 million reads per sample.

The initial bioinformatics analyses, including read filtering, alignment, transcript assembly, gene quantification, and functional enrichment (GO and KEGG), were carried out by Novogene Europe. Raw sequencing reads were quality-filtered using fastp (Chen et al. 2018) to remove adapters and low-quality reads. Clean reads were aligned to the *Saccharomyces cerevisiae* W303 reference genome (JRIU00000000) using HISAT2 (v2.2.1) (Kim et al. 2019). Transcript assembly was performed using StringTie (v2.2.3) (Pertea et al. 2015), and gene-level expression was quantified using featureCounts (v2.0.6) (Liao et al. 2014). Expression values were normalized as FPKM (fragments per kilobase of transcript per million mapped reads) (Trapnell et al. 2010) to perform principal component and cluster analyses.

Differential expression analyses were performed on raw read counts using the DESeq2 tool (v1.42.0) (Love et al. 2014). *P*-values were adjusted using the Benjamini–Hochberg false discovery rate method. Genes with an adjusted *P* value (*P*adj) ≤ 0.05 were considered significantly differentially expressed.

Gene Ontology (GO) enrichment analysis was performed using the clusterProfiler (v4.8.1) R package (Wu et al. 2021), correcting for gene length bias. Enriched GO terms and pathways were considered significant at *P*adj ≤ 0.05.

Heatmaps, Venn diagrams, and GO enrichment analyses were generated using the NovoMagic platform (https://www.novogene.com/euen/novomagic-online-rna-seq-bioinformatics-analysis-tool/).

Spearman's rank correlation was used to assess the relationship between fold-change magnitude and gene distance from the nearest telomere. Enrichment of Rap1 binding sites among shared differentially expressed genes (DEGs) was assessed using the hypergeometric test, with the total gene set defined as all genes detected by RNA-seq (*n* = 5,696). Rap1 DNA-binding targets were obtained from the YEASTRACT database (Teixeira et al. 2023). The same test was used to assess overlap with genes affected by Rap1 depletion (Kalra et al. 2023), using their total gene set (*n* = 5,088). Paired differences in fold-change magnitude were assessed using the Wilcoxon signed-rank test.

## Results

To test whether long telomeres influence gene expression, RNA sequencing was performed on diploid yeast strains that had different telomere lengths and were derived from a common lineage. Four diploid genotypes were analyzed: a wild-type control (*WT/WT*), a heterozygous diploid carrying a mutation in the telomere length regulator *RIF2* which resulted in telomere elongation (*WT/rif2*Δ), and 2 genetically wild-type diploids with long VIR telomeres (*WT/WT_long1* and *WT/WT_long2*). All 4 diploids were generated by crossing haploid strains to the same wild-type haploid strain (Fig. 1a). The *WT_long1* and *WT_long2* haploids were derived in a previous study through successive rounds of mating and meiosis that transmitted long VIR telomeres from a *rif2*Δ ancestor (Sidarava et al. 2025) (Fig. 1a). As a result, *WT/rif2*Δ, *WT/WT_long1*, and *WT/WT_long2* are from a single lineage in which long telomeres were originally caused by a *rif2*Δ mutation.

**Fig. 1.**
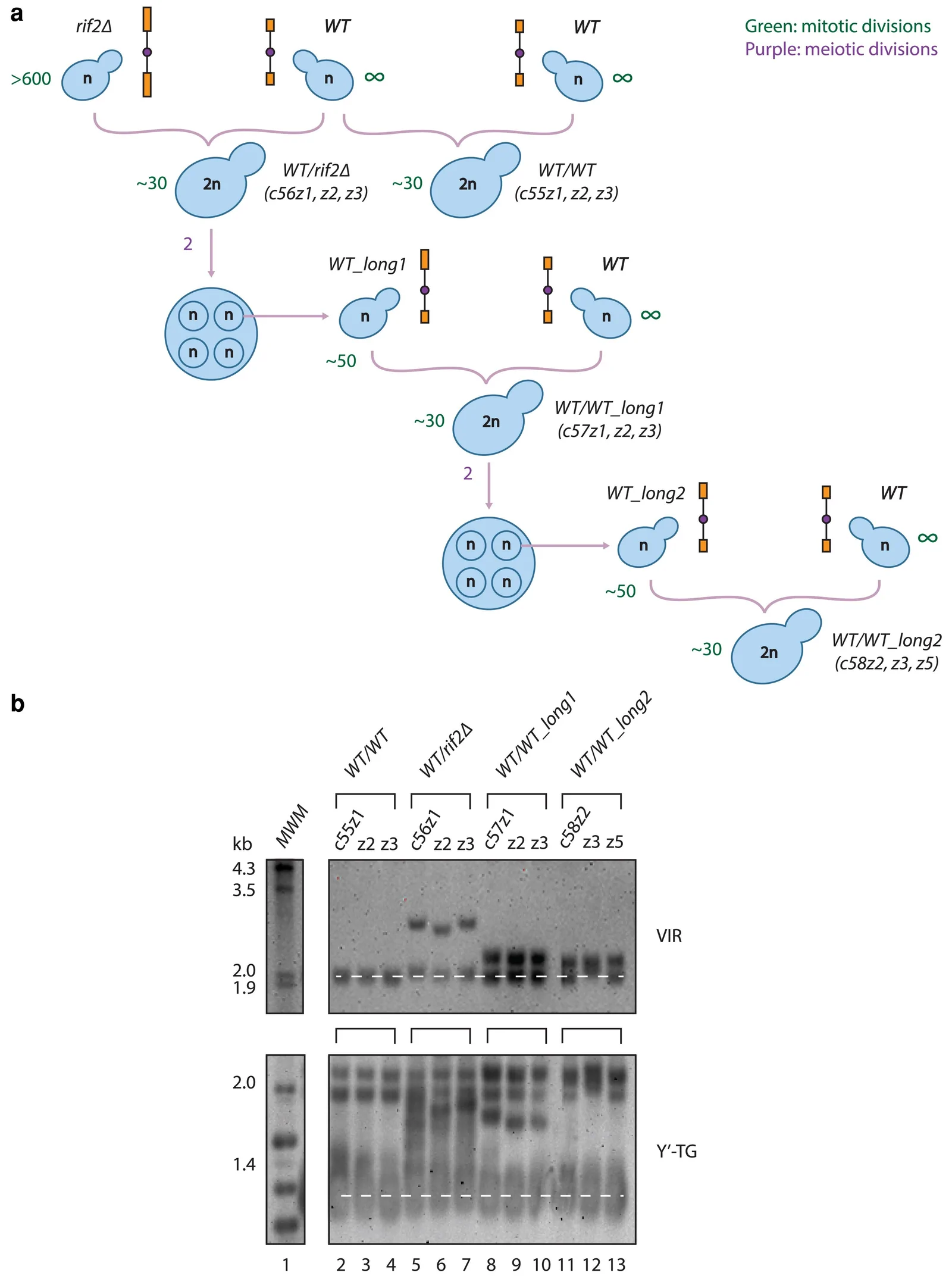
Origin and telomere length of strains used for transcriptome analysis. a) Lineage of strains analyzed. A *rif2*Δ haploid was crossed to a wild-type haploid strain to generate a *WT/rif2*Δ diploid, which was sporulated to produce meiotic progeny. From this, a wild-type haploid carrying long VIR telomeres (*WT_long1*) was isolated. *WT_long1* was crossed to the wild-type strain to generate a *WT/WT_long1* diploid, which was subsequently sporulated to yield a second haploid with long VIR telomeres (*WT_long2*). *WT_long2* was crossed to the wild-type strain to generate the *WT/WT_long2* diploid. Biological replicates for each diploid genotype examined are indicated in parentheses. Estimated numbers of mitotic and meiotic divisions undergone by haploid and diploid cells are indicated in green and purple, respectively, and were determined as described previously (Sidarava et al. 2025). The cells went through 4 further divisions in liquid culture prior to DNA extraction. (Adapted from Sidarava et al. 2025). b) Telomeric Southern blots. Three biological replicates for each genotype are shown. The *WT/WT* diploids served as controls. Telomere length was measured using VIR and Y′-TG probes. Dashed white line indicates normal telomere length.

Diploids were chosen for transcriptome analysis rather than haploids to minimize potential confounding effects of recessive background mutations that might be expressed in haploid cells (Szafraniec et al. 2003). Furthermore, diploidy is the primary state of *S. cerevisiae* during vegetative growth in nature (Peter et al. 2018). In addition, diploid cells more closely reflect the chromosomal state of human somatic cells.

For each class of diploid, 3 biological replicates were obtained by isolating 3 independent zygotes from each cross. Diploid clones were expanded from zygotes, on plates and in liquid before cultures were split: half was used for DNA extraction and telomere length analysis, and the other half for RNA extraction and transcriptome analysis. This approach ensured that the transcriptome measured from each culture could be directly matched to telomere length measured from the same culture.

*WT/WT* strains had normal VIR telomere lengths: terminal restriction fragments were ∼2.0 kb, of which the TG_1−3_ tract is ∼340 bp (Sholes et al. 2022) and the remainder is subtelomeric sequence (Fig. 1b). Consistent with our earlier work, 2 sets of VIR telomeres could be detected in *WT/rif2*Δ, *WT/WT_long1*, and *WT/WT_long2* strains, the shorter ones presumably derived from the wild-type parent and the longer ones from long-telomere parents (Sidarava et al. 2025). *WT/rif2*Δ cells had the longest VIR telomeres (∼3.1 kb), while in *WT/WT_long1* and *WT/WT_long2* strains, the long VIR telomeres were 2.4 and 2.3 kb long, respectively. We estimate that there were 166 cell divisions between the segregation of the *rif2*Δ mutation and a long VIR telomere, and the cells in the *WT/WT_long2* cultures used for RNA extraction (Fig. 1a). During these divisions, VIR telomeres shortened gradually (Sidarava et al. 2025), most likely due to the end replication problem and reduced telomerase-mediated elongation. While we focused on VIR as a representative telomere, we note that other individual telomeres in this lineage were likely also elongated and inherited, though not directly measured in this study.

A Y′-TG probe was also used to examine average length of Y′ telomeres. Consistent with what we previously reported (Sidarava et al. 2025), the Y′-TG probe showed that *WT/rif2*Δ diploid cells had long Y′-telomeres (due to haploinsufficiency, or because it takes time for long telomeres to normalize in length). The *WT/WT*, *WT/WT_long1*, and *WT/WT_long2* cells had essentially normal length Y′-telomeres, but the *WT/WT_long1*, and *WT/WT_long2* cells had at least one long telomere (VIR). Overall, our Southern blots confirmed that the cultures we had harvested for transcriptome profiling contained the expected telomere lengths (of VIR and Y′).

To determine whether strains with long VIR telomeres shared transcriptional profiles, RNA sequencing data were analyzed. First, principal component analysis (PCA) was performed to assess variation in global gene expression and the similarity between samples across all classes of diploids (Supplementary Fig. 1). Expression values were normalized using FPKM (fragments per kilobase of transcript per million mapped reads), which accounts for transcript length and sequencing depth. Reassuringly, most biological replicates clustered together, indicating consistency of gene expression profile with groups of clones. However, sample c56z1 (*WT/rif2*Δ) showed strong deviation from its group, separating clearly from all other samples along the first principal component. Standard sequencing quality metrics, including clean read percentage, GC content distribution, error rate, and mapping rate, were comparable in c56z1 and the other samples, suggesting that the deviation reflects biological rather than technical variability. Because the inclusion of this sample disproportionately influenced the variance structure used for group comparisons, it was excluded from the primary analyses presented here. All analyses that follow were also repeated with c56z1 included (Supplementary File 2), and similar results obtained.

To identify transcriptional changes associated with long telomeres, pairwise differential expression analyses were performed between each long-telomere strain and the *WT/WT* control using DESeq2 (see Methods). For each gene, fold changes and adjusted *P*-values were calculated. Lists of significant DEGs (adjusted *P* ≤ 0.05) for all 3 comparisons are provided in Supplementary File 1.

Inspection of individual DEGs by pairwise comparison revealed upregulation of diverse transporter genes, including phosphate, amino acid, and sugar transporters (Supplementary File 1). In addition, the starvation marker *YGP1* was upregulated (Destruelle et al. 1994), while ribosomal and mitochondrial genes were downregulated. This pattern is consistent with a starvation-like transcriptional response (Wu et al. 2004; Petti et al. 2011).

Histone genes from all 4 core histone families (H2A, H2B, H3, and H4) were downregulated in strains that inherited long telomeres (*WT/WT_long1* and *WT/WT_long2*; fold change 0.63 to 0.85×; Supplementary File 1), as also seen under nutrient limitation (Chatzitheodoridou et al. 2024).

To compare transcriptional patterns across strains, all significant DEGs (adjusted *P* value ≤ 0.05) from the 3 pairwise comparisons were combined and subjected to hierarchical clustering. The resulting heatmap showed clear separation between *WT/WT* samples and all long-telomere strains, indicating that long telomeres induce a distinct transcriptional profile (Fig. 2). Replicates of *WT/WT_long1* and *WT/WT_long2* generally clustered together, supporting the reproducibility of inherited long-telomere effects across generations. One *WT/WT_long*1 replicate clustered closer to *WT/rif2*Δ in the heatmap, consistent with its position in the PCA (Supplementary Fig. 1).

**Fig. 2.**
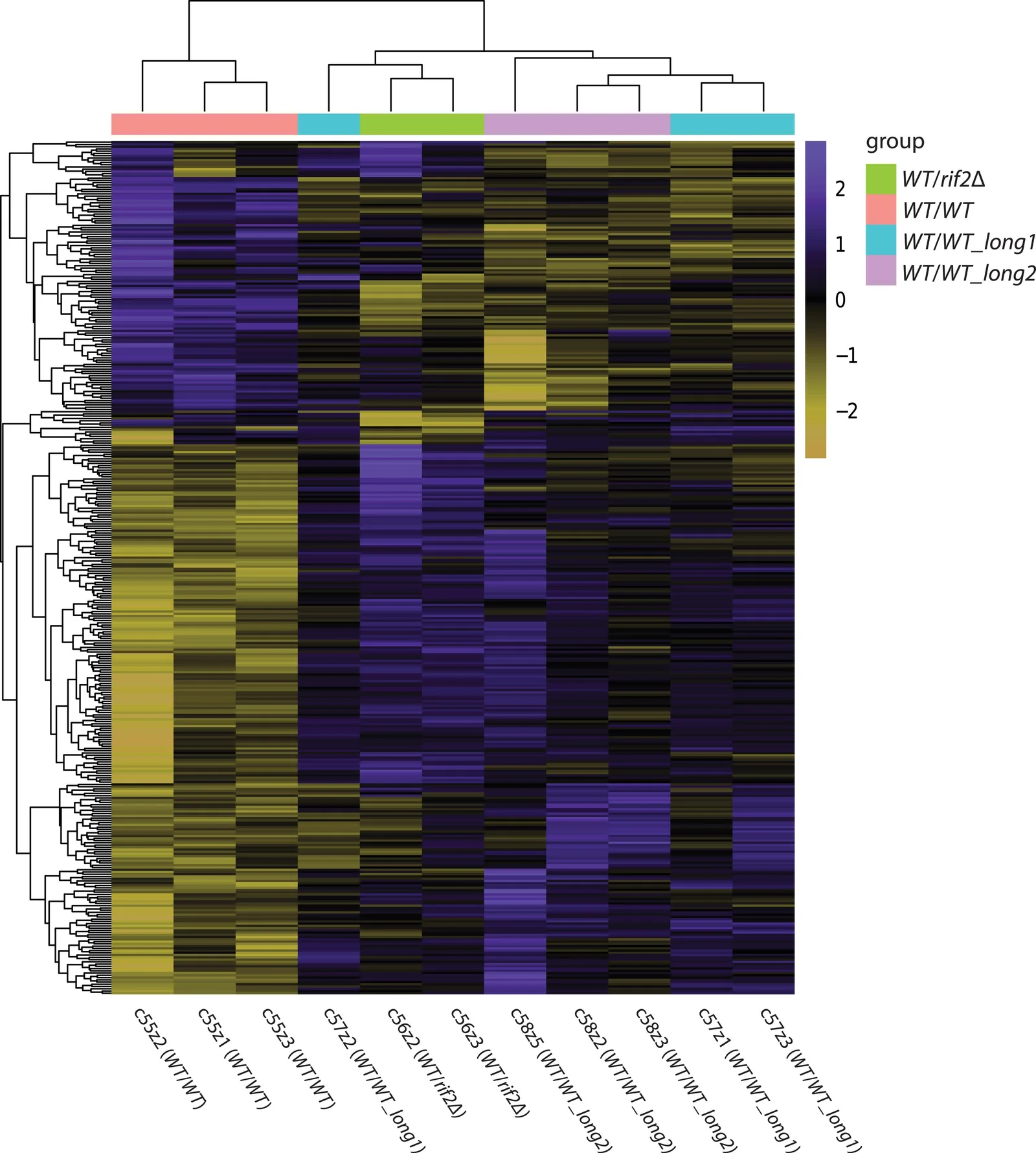
Long telomere strains (where long telomeres are either inherited or mutation-induced) share a distinct transcriptional profile from wild type. Heatmap of combined significant DEGs (adjusted *P* ≤ 0.05) from pairwise comparisons between long telomere strains and the wild type. Each column represents a sample (replicate), and each row represents a gene. Expression values were normalized as FPKM. For visualization, values were also row-normalized using Z-scores (color intensity reflects each gene's expression relative to its mean across all samples). Blue color indicates upregulation and yellow color indicates downregulation.

The overlap between DEGs identified in the 3 pairwise comparisons was visualized using a Venn diagram (Fig. 3). Fifty-seven DEGs were shared across all long-telomere conditions, defining a common transcriptional response associated with long telomeres (Table 2). In addition, each comparison contained a substantial number of unique DEGs (for example, 103 genes unique to *WT/WT_long2* vs *WT/WT*), suggesting that long telomeres might also produce strain- or generation-specific transcriptional effects.

**Fig. 3.**
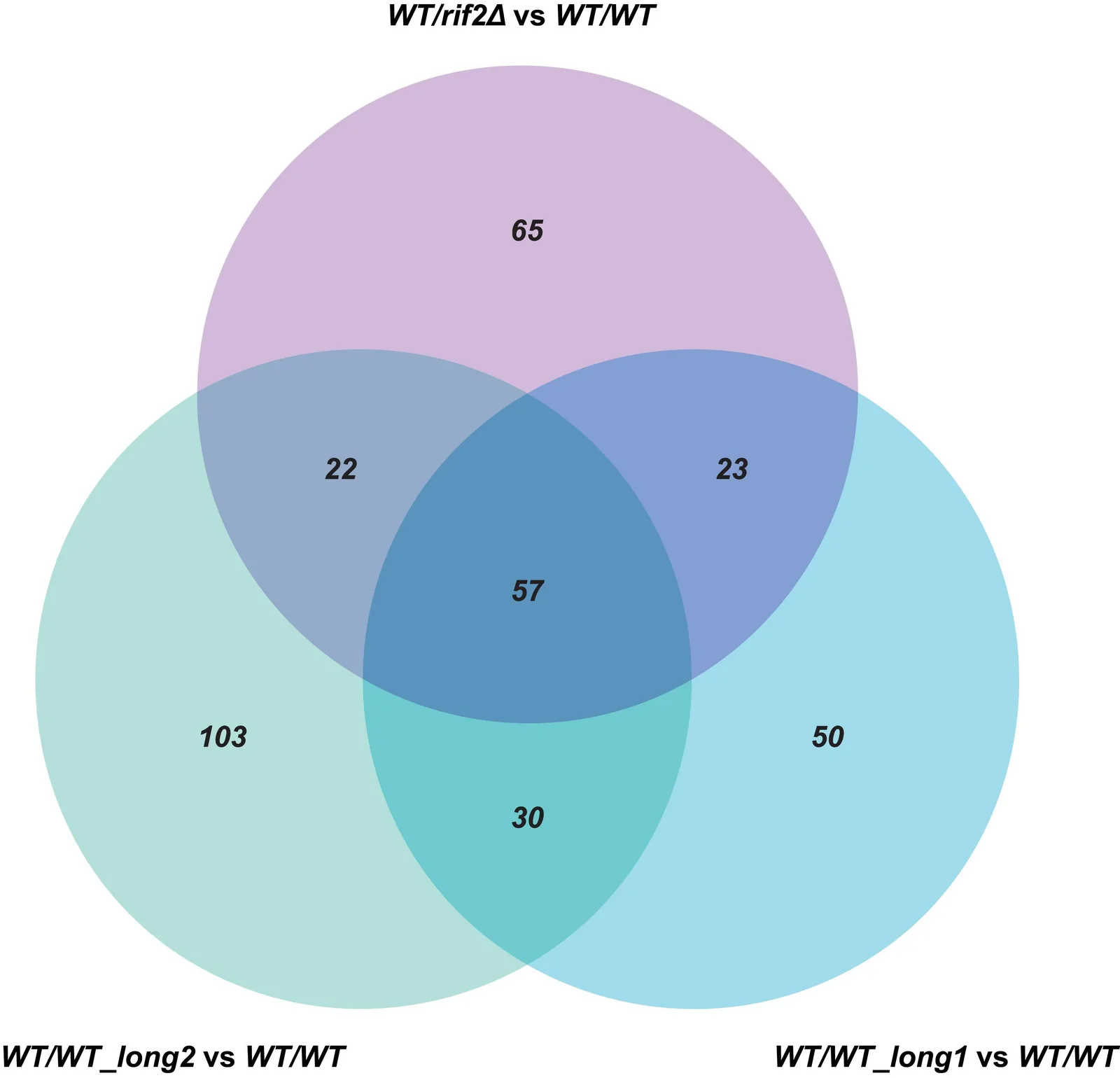
Long-telomere strains share a core set of differentially expressed genes. Venn diagram illustrating the overlap between significantly differentially expressed genes (adjusted *P* ≤ 0.05) from 3 pairwise comparisons: *WT/WT_long1* vs *WT/WT* (blue), *WT/WT_long2* vs *WT/WT* (green), and *WT/rif2*Δ vs *WT/WT* (purple). Numbers in the diagram indicate DEGs unique to a given comparison (nonoverlapping regions) or shared between comparisons (overlapping regions).

**Table 2.** Genomic position and Rap1-binding status of the 57 DEGs.

Gene ID	Gene standard name and function	Chromosome number	Distance from telomere (kb)	Binds Rap1?
*YML128C*	*MSC1*—Meiotic sister chromatid recombination protein 1	XIII	15	No
*YDR536W*	*STL1*—Sugar transporter	IV	23	No
*YML123C*	*PHO84*—Inorganic phosphate transporter	XIII	24	Yes
*YKL216W*	*URA1*—Dihydroorotate dehydrogenase	XI	25	No
*YNR067C*	*DSE4*—Glucan endo-1,3-beta-D-glucosidase involved in cell separation after cytokinesis	XIV	29	No
*YDL237W*	*AIM6*—Altered inheritance of mitochondria protein 6	IV	30	No
*YLL055W*	*YCT1*—High-affinity cysteine transporter	XII	30	Yes
*YBR286W*	*APE3*—Vacuolar aminopeptidase Y	II	39	No
*YPL265W*	*DIP5*—Dicarboxylic amino acid permease	XVI	41	No
*YDL223C*	*HBT1*	IV	57	No
*YHR202W*	*SMN1*—Putative AMP hydrolase	VIII	60	No
*YHL016C*	*DUR3*—Urea active transporter	VIII	72	Yes
*YKL195W*	*MIA40*—Import and assembly protein in mitochondrial intermembrane space	XI	76	No
*YGR260W*	*TNA1*—High-affinity nicotinic acid transporter	VII	78	Yes
*YCR068W*	*ATG15*—Putative lipase	III	79	Yes
*YDL210W*	*UGA4*—GABA-specific permease	IV	84	No
*YKL187C*	*FAT3*—Fatty acid transporter	XI	89	No
*YCR061W*	*YCV1*—Uncharacterized membrane protein YCR061W	III	91	No
*YDL204W*	*RTN2*—Reticulon-like protein 2	IV	94	Yes
*YOL119C*	*MCH4*—Probable transporter	XV	95	Yes
*YDR492W*	*IZH1*—ADIPOR-like receptor	IV	97	No
*YJL172W*	*CBPS*—Carboxypeptidase S	X	98	No
*YFL016C*	*MDJ1*—DnaJ homolog 1, mitochondrial	VI	104	Yes
*YPR158W*	*CUR1*—Curing of [URE3] protein 1	XVI	105	No
*YDR483W*	*KRE2*—Glycolipid 2-alphamannosyltransferase	IV	110	No
*novel.119*	*TPO3*—Polyamine transporter 3	XVI	111	No
*YJL163C*	*YJQ3*—Uncharacterized membrane protein YJL163C	X	112	No
*YML075C*	*HMDH1*–3-hydroxy-3-methylglutarylcoenzyme A reductase 1	XIII	115	No
*YMR266W*	*RSN1*—Uncharacterized protein	XIII	126	Yes
*YBR235W*	*VHC1*—Vacuolar cation-chloride cotransporter 1	II	127	No
*YNL268W*	*LYP1*—Lysine-specific permease	XIV	138	Yes
*YCR017C*	*CWH43*	III	144	No
*YGL186C*	*TPN1*—Vitamin B6 transporter	VII	151	No
*YIL030C*	*DOA10*—ERAD-associated E3 ubiquitin-protein ligase	IX	154	No
*YCR021C*	*HSP30*–30 kDa heat shock protein	III	160	No
*YNL237W*	*YTP1*—Protein	XIV	205	No
*YHR096C*	*HXT5*—Probable glucose transporter	VIII	268	Yes
*YNL195C*	*YNT5*—Uncharacterized protein YNL195C	XIV	271	No
*YLR069C*	*EFGM*—Elongation factor G, mitochondrial	XII	271	No
*YKL034W*	*TUL1*—Transmembrane E3 ubiquitinprotein ligase 1	XI	295	No
*YOR002W*	*ALG6*—Dolichyl pyrophosphate Man9GlcNAc2 alpha-1,3glucosyltransferase	XV	329	No
*YNL160W*	*YGP1*—Protein YGP1	XIV	336	No
*YMR034C*	*RCH1*—Solute carrier	XIII	339	No
*YBR054W*	*YRO2*	II	343	No
*YLR299W*	*ECM38*—Glutathione hydrolase proenzyme	XII	352	Yes
*YNL142W*	*MEP2*—Ammonium transporter	XIV	357	Yes
*YDL054C*	*MCH1*—Probable transporter	IV	360	Yes
*YBR067C*	*TIP1*—Temperature shock-inducible protein 1	II	372	No
*YBR072W*	*HSP26*—Small heat shock protein with chaperone activity	II	382	Yes
*YGL055W*	*OLE1*—Delta(9) fatty acid desaturase	VII	399	Yes
*YOR187W*	*EFTU*—Elongation factor Tu, mitochondrial	XV	407	No
*YGL008C*	*PMA1*—Plasma membrane ATPase 1	VII	479	Yes
*YGR060W*	*ERG25*—C-4 methylsterol oxidase	VII	480	Yes
*YGL006W*	*ATC2*—Calcium-transporting ATPase 2	VII	486	Yes
*YLR207W*	*HRD3*—ERAD-associated E3 ubiquitin–protein ligase component	XII	522	No
*YDR135C*	*YCF1*—Metal resistance protein	IV	723	Yes
*YDR205W*	*MSC2*—Probable zinc transporter	IV	772	No

To explore whether the set of 57 DEGs represented specific functional categories, Gene Ontology enrichment analysis was performed. This revealed significant overrepresentation of transport-related categories (Supplementary Fig. 2), consistent with the transporter induction observed in the individual comparisons. No other functional categories showed strong enrichment.

Interestingly, subtelomeric regions are enriched in stress-responsive and transporter genes (Ai et al. 2002; Brown et al. 2010). Thus, we hypothesized that the change in expression of transporters and other shared DEGs could be explained by changes in subtelomeric chromatin induced by long telomeres. For example, long telomeres could affect TPE, whereby the expression of genes in proximity to long telomeres is repressed. However, only 7 of the 57 shared DEGs were located within 30 kb of a chromosome end (Table 2), and no significant correlation was observed between the fold change and distance from telomeres in any strain (Fig. 4). Together, these observations argue against telomere position effect as the primary driver of the shared transcriptional response.

**Fig. 4.**
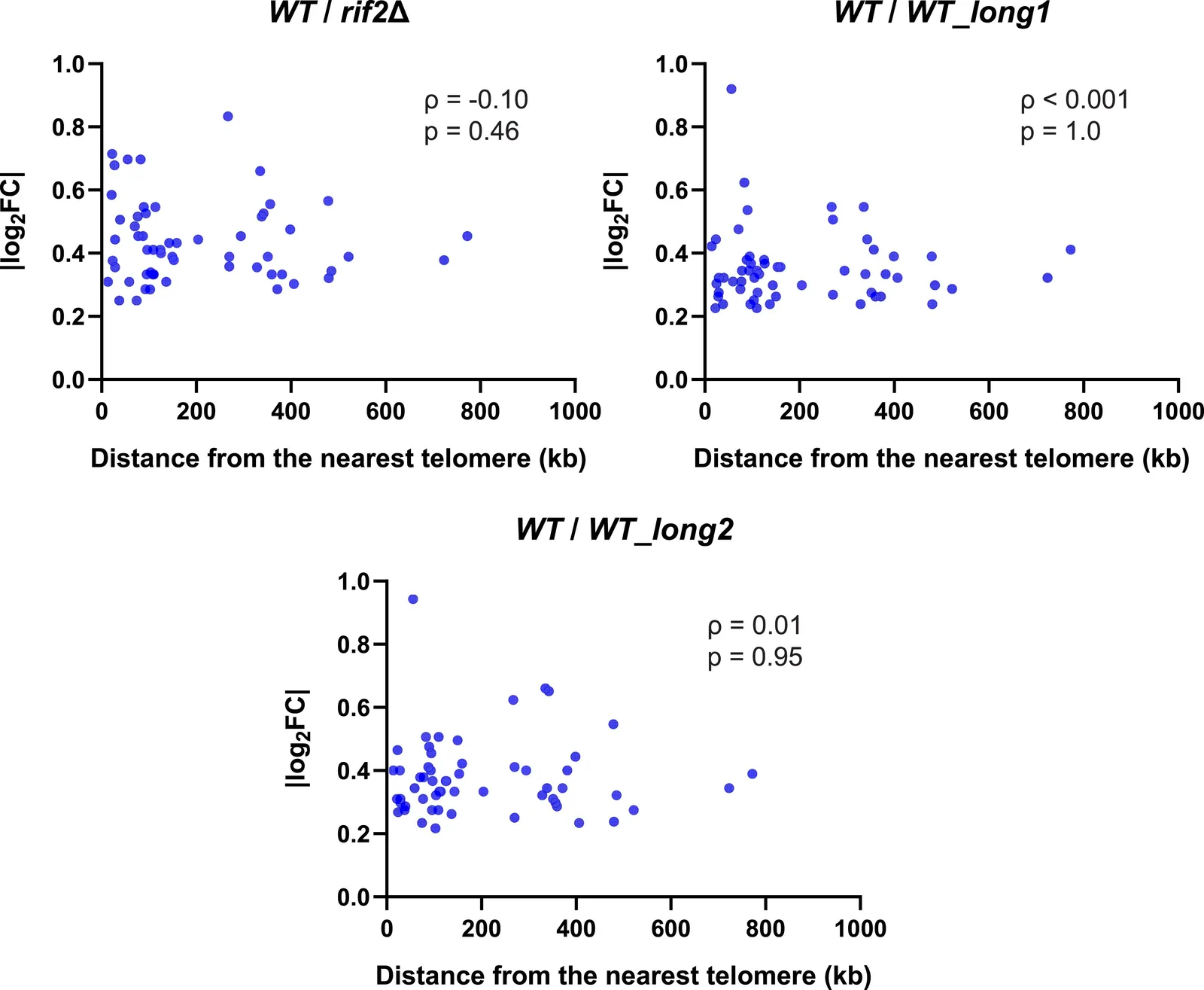
The observed transcriptional changes do not correlate with the distance from the nearest telomere. Scatter plots showing the relationship between fold-change magnitude (|log_2_FC|) and distance from the nearest telomere (kb) for the 57 DEGs in *WT/rif2*Δ, *WT/WT_long1*, and *WT/WT_long2* strains. Each point represents a gene. Spearman's rank correlation was used to assess the relationship between variables, with the corresponding ρ and *P* values indicated on each plot.

One possible explanation for the observed genome-wide changes in gene expression is the sequestration of transcriptional regulators away from their internal genomic targets by telomeric DNA. Rap1 is a good candidate of such a regulator: in addition to binding telomeric DNA, Rap1 is a transcriptional regulator with 1,582 documented DNA-binding targets across the genome (YEASTRACT [Yeast Search for Transcriptional Regulators And Consensus Tracking] database [Teixeira et al. 2023]). If an elongated telomere reduces Rap1 occupancy at non-telomeric promoters, this could lead to misregulation of those genes. To test this hypothesis, the 57 shared DEGs were cross-referenced with YEASTRACT annotations: 20 of the 57 showed documented Rap1 DNA-binding evidence (Table 2). This proportion is higher than expected by chance (hypergeometric test, *P* ≈ 0.02, based on 5,696 genes detected by RNA-seq, of which 1,285 bind Rap1), consistent with a model in which long telomeres alter Rap1 availability at some of its internal targets and thereby contribute to the shared transcriptional response. The Sir2/3/4 complex, which also functions at both telomeres and internal loci, is another candidate for such redistribution. However, none of the 57 shared DEGs overlapped with SIR-dependent genes identified at native telomeres (Ellahi et al. 2015), arguing against SIR-mediated silencing as a contributor to the observed response. In addition, because neither subtelomeric localization nor Rap1/SIR binding accounts for all shared DEGs, additional regulatory mechanisms or indirect downstream effects are likely to also contribute to the transcriptional consequences of telomere elongation.

Previous work has shown that partial depletion of Rap1 using a doxycycline repressible system results in widespread transcriptional changes, with distinct gene sets responding at different levels of Rap1 abundance (Kalra et al. 2023). However, overlap between our 57 DEGs and genes responsive to Rap1 depletion was inconsistent across depletion levels (Supplementary Table 1). At approximately 60%, 40%, and 20% of normal Rap1 levels, the Kalra et al. dataset identified 1,221, 1,920, and 2,341 affected genes, respectively, of which 17, 26, and 24 overlapped with our 57 DEGs. Only the intermediate depletion level showed significant enrichment (hypergeometric test: *P* = 0.188, 0.022, and 0.324, respectively; total number of genes analyzed by Kalra et al. – 5088). This may reflect differences between partial Rap1 loss and partial redistribution of Rap1 predicted by our model. Direct tests of Rap1 occupancy at promoters in long-telomere strains would be needed to confirm redistribution.

Since telomere length decreased between *WT/rif2*Δ cells and *WT/WT_long1* cells and further in *WT/WT_long2* cells (Fig. 1b), we hypothesized that the magnitude of changes in levels of DEGs would change accordingly. The changes for each gene were compared between pairs of strains (Fig. 5). Fold changes were significantly reduced from *WT*/*rif2*Δ to *WT*/*WT_long1* (Wilcoxon signed-rank test, *P* < 0.0001; median Δ|log_2_FC| = 0.077) but did not decrease further between *WT*/*WT_long1* and *WT*/*WT_long2* (slight increase in magnitude, *P* = 0.0051; median Δ|log_2_FC| = 0.024).

**Fig. 5.**
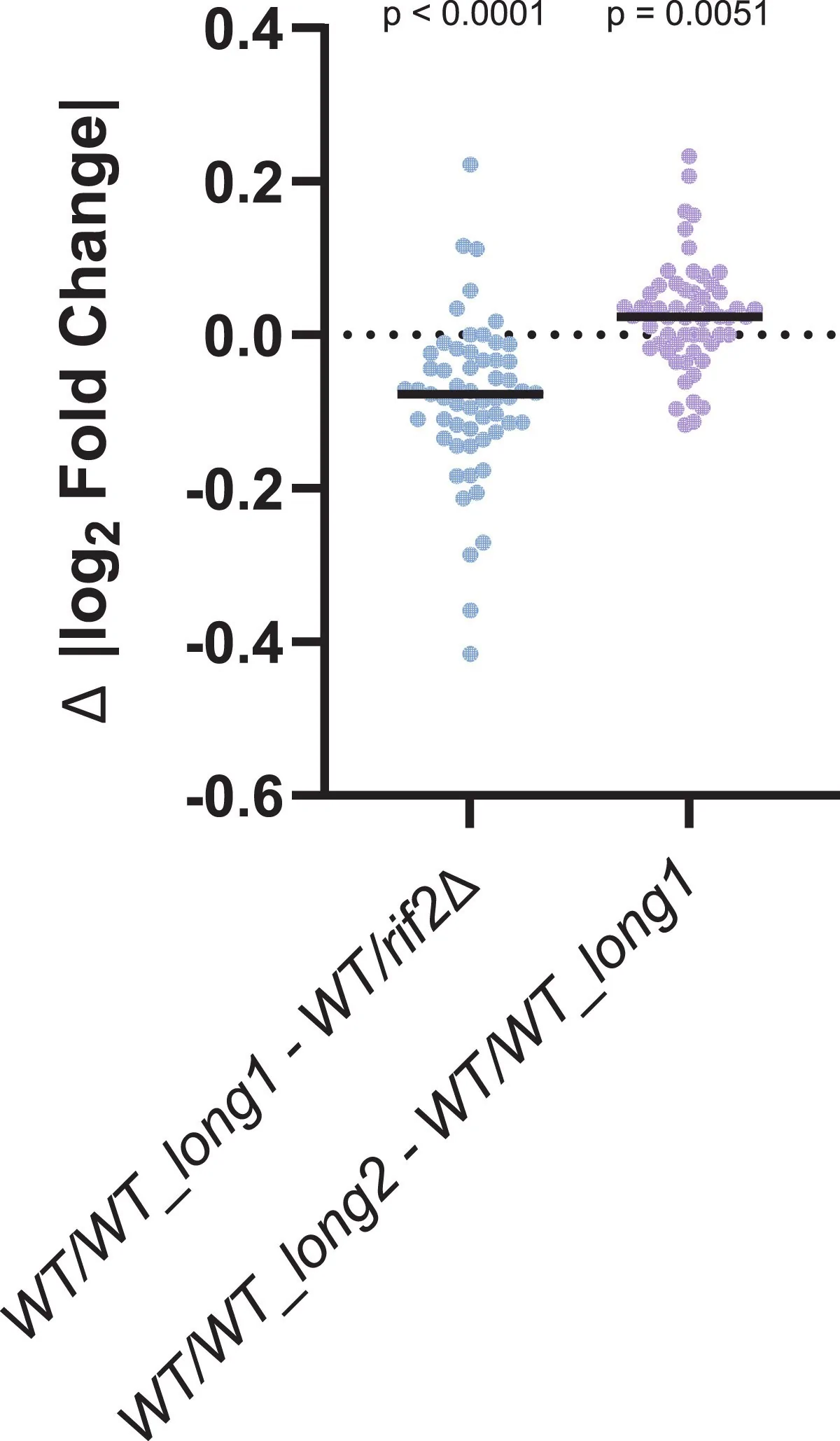
Per-gene changes in |log_2_FC| across long-telomere strains. Paired differences in absolute log_2_ fold-change (|log_2_FC|) values were calculated for each of the 57 shared DEGs between *WT/WT_long1* and *WT/rif2*Δ, and between *WT/WT_long2* and *WT/WT_long1*. Each point represents one gene. The dashed line indicates zero difference. Solid lines indicate medians. A Wilcoxon signed-rank test was used to assess whether paired |log_2_FC| differences were significantly different from zero; *P*-values are shown.

This pattern is broadly consistent with the telomere length data. In *WT/rif2*Δ diploids, the *rif2*Δ haploid parent contributed elongated telomeres at all 32 chromosome ends, so approximately half of all telomeres in the diploid are expected to be long, with VIR telomeres reaching ∼3.1 kb. In contrast, *WT/WT_long1* and *WT/WT_long2* diploids carried normal Y′-telomeres and presumably only a subset of long telomeres, including VIR, which were detectable but shorter than in the *WT/rif2*Δ diploid (∼2.4 and ∼2.3 kb, respectively) (Fig. 1b). The large reduction in total long-telomere burden between *WT/rif2*Δ and *WT/WT_long1* likely contributes to the corresponding drop in transcriptional response, while the similar VIR lengths in *WT/WT_long1* and *WT/WT_long2* are consistent with their comparable expression profiles. These observations are consistent with a model in which the transcriptional response scales with the amount of telomeric DNA available to sequester transcriptional regulators.

## Discussion

RNA-seq analysis of 3 generations of yeast strains, in which long telomeres were initially generated by a *rif2*Δ mutation and subsequently inherited by wild-type cells, revealed a genome-wide transcriptional response to long telomeres. A core set of 57 genes was consistently differentially expressed across all long-telomere strains. Upregulated genes were enriched for membrane transporters, while downregulated genes included several genes involved in mitochondrial translation and import. This pattern resembles transcriptional responses reported under nutrient deprivation (Wu et al. 2004).

We considered whether the shared transcriptional response could be explained by telomere position effect (subtelomeric gene silencing). Only a small fraction of the shared DEGs identified here were located within 30 kb of chromosome ends, and fold-change magnitude did not correlate with distance from telomeres. This argues against altered subtelomeric silencing as the primary driver of the shared transcriptional response to telomere elongation. In contrast, our data are consistent with a model in which long telomeres influence transcription through redistribution of telomere-binding transcriptional regulators, such as Rap1, away from their internal genomic targets. The enrichment of Rap1 binding sites among shared DEGs supports this model, although other telomere-binding proteins that also affect gene expression elsewhere in the genome may contribute similarly. Consistent with this model, the strongest changes in gene expression were observed in *WT/rif2*Δ strains, which had the longest telomeres, followed by smaller changes in *WT/WT_long1* and *WT/WT_long2*.

To estimate the potential redistribution of telomere-binding proteins quantitatively, we used Rap1 as an example. Rap1 is present at approximately 4,000 to 5,000 molecules per haploid cell (Ho et al. 2018), and approximately double this in diploids. At telomeres, Rap1 binds approximately once per 18 bp of TG_1–3_ repeat sequence (Gilson et al. 1993), with cooperative binding at adjacent sites further increasing effective affinity (Giraldo and Rhodes 1994). Attributing the difference between the ∼2.0 kb (*WT/WT*) and ∼3.1 kb (*WT/rif2*Δ) VIR terminal restriction fragments to additional TG_1−3_ repeats, the VIR TG tract is estimated to increase from ∼340 bp to ∼1,440 bp (3.2-fold), sequestering 61 additional Rap1 molecules at that end. Assuming comparable elongation at the 32 chromosome ends contributed by the *rif2*Δ parent in *WT/rif2*Δ diploids, this gives ∼2,000 additional Rap1 molecules sequestered genome-wide, which is roughly 20% to 25% of the total diploid Rap1 pool. For *WT/WT_long1* and *WT/WT_long2*, we expect fewer telomeres to be elongated, and the total additional sequestration to be smaller. These estimates suggest that the scale of Rap1 redistribution in the strains with long telomeres is sufficient to reduce Rap1 availability at internal promoters.

An alternative, non-exclusive explanation for the transcriptional response to long telomeres is that long telomeres impose a metabolic burden on the cell. Replication and maintenance of elongated telomeric DNA could increase demand for nucleotides or energy, leading to a physiological state that resembles nutrient limitation and triggers compensatory transporter induction. A link between nutrient sensing and telomere maintenance has been reported: inhibition of TOR signaling has been linked to telomere shortening, suggesting crosstalk between metabolic state and telomere regulation (Ungar et al. 2011; Kupiec and Weisman 2012). However, we did not detect changes in expression of TOR pathway components in our strains. Additionally, in the most extreme case (*WT/rif2*Δ), we estimate that elongated telomeres contribute approximately 35 kb (about 1.1 kb extra per telomere) of additional telomeric DNA, representing less than 0.15% of the diploid genome. Thus, it is unlikely that such a small increase in replication load is sufficient to alter cellular metabolism.

Since in our experiments the diploid wild-type strains with long telomeres were derived from a *WT/rif2*Δ diploid, we considered whether the transcriptional response might reflect long-lasting telomere-length-independent consequences of the *rif2*Δ mutation. However, the same core transcriptional signature (57 DEGs) was observed in wildtype segregants with long telomeres after meiosis and subsequent propagation, indicating that long telomeres, rather than the *rif2*Δ mutation itself, are the primary driver of the observed expression changes.

Overall, the magnitude of gene expression changes between long-telomere strains and wild-type controls was modest (typically <2-fold for the 57 DEGs). It is well established, however, that even modest changes in gene dosage can have significant phenotypic consequences. In human aneuploidies such as Down syndrome, a 1.5-fold increase in chromosome 21 gene copy number and presumably gene expression is sufficient to cause this complex multi-system disorder (Antonarakis 2017). Dosage-sensitive genes more broadly associate with disease phenotypes including cardiac defects, cancers, and neuropsychiatric disorders (Rice and McLysaght 2017). In *S. cerevisiae*, subtle shifts in gene expression can influence cellular behavior under specific environmental conditions (Rest et al. 2013; Keren et al. 2016), or in combination with other mutations. It should also be noted that budding yeast exhibits post-transcriptional buffering that can partially compensate for changes in mRNA levels (Blevins et al. 2019; Teyssonniere et al. 2024), potentially mitigating the functional consequences of the transcriptional changes we report.

Telomere length-associated effects on gene expression have been reported in other eukaryotic systems. In human cells, telomere position effect has been documented both locally (Baur et al. 2001) and over long genomic distances (Kim and Shay 2018). At the same time, multiple telomeric proteins, including RAP1 and other shelterin components, have been shown to bind internal genomic sites and regulate transcription (Yang et al. 2011). However, whether telomere length modulates this regulation genome-wide has not been tested. Our findings demonstrate that long telomeres induce a genome-wide transcriptional response in yeast, with Rap1 targets enriched among the affected genes. Whether similar length-dependent transcriptional responses occur in mammalian cells, and whether they involve redistribution of telomere-associated factors, remains an important question for future study.

## Supplementary Material

iyag174_Supplementary_Data

## Data Availability

Raw RNA-seq reads and raw gene counts for all samples have been deposited in NCBI's Gene Expression Omnibus (Edgar et al. 2002) and are accessible through GEO Series accession number GSE327262 (https://www.ncbi.nlm.nih.gov/geo/query/acc.cgi?acc=GSE327262). Differentially expressed gene lists are provided in Supplementary Files 1 and 2. All strains used in this study are available upon request. Supplemental material available at GENETICS online.
