# Supplementary material for: Inherited long telomeres induce a genome-wide transcriptional response in budding yeast
Source: Genetics. 2026 Jul 3;234(2):iyag174. doi: 10.1093/genetics/iyag174 (PMC13643770; doi:10.1093/genetics/iyag174)
Supplement: Supplementary file 1 [file iyag174_supplementary_data.zip › Figure_S1_GENETICS-2026-309600.pdf]

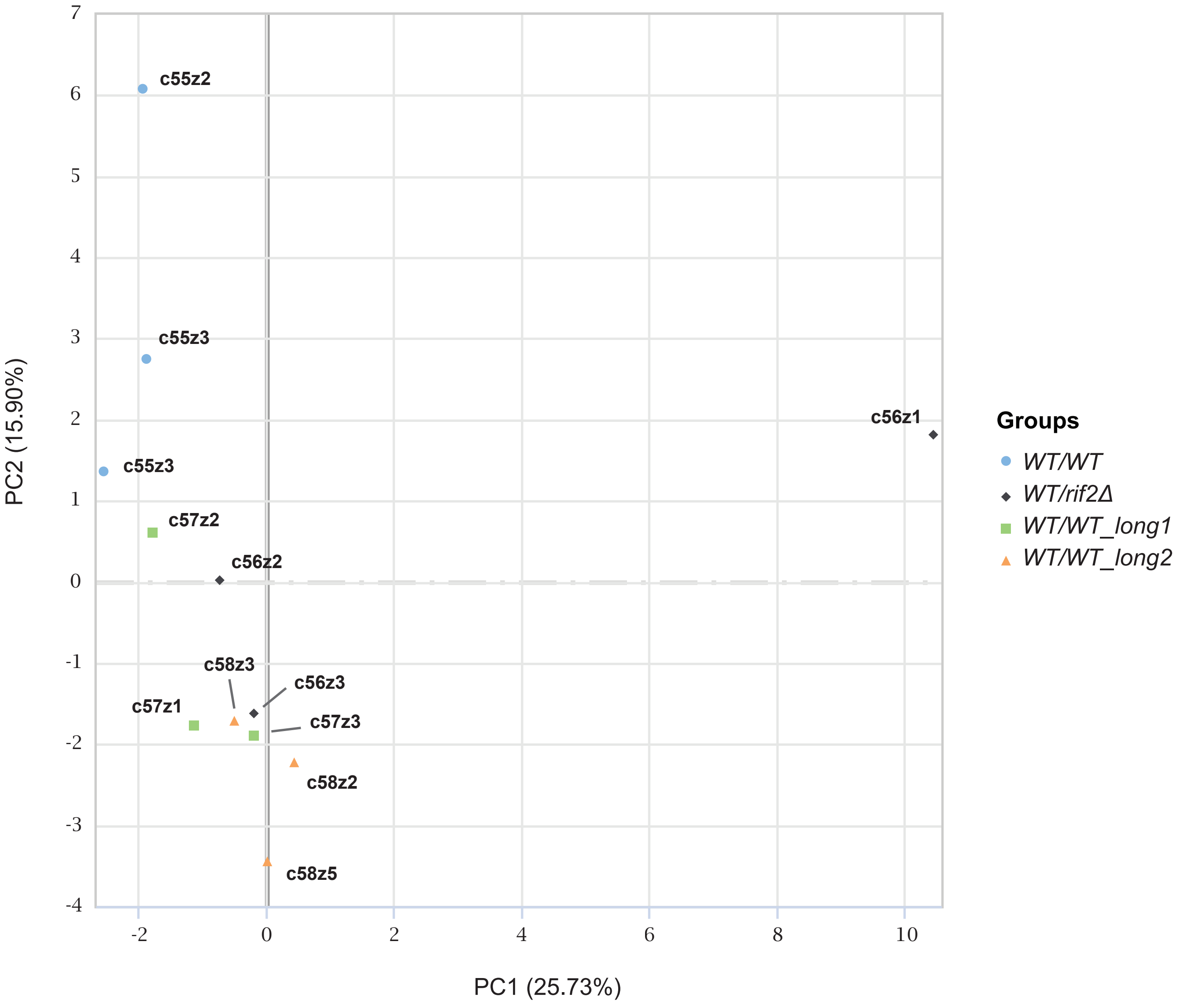

PC2 (15.90%)
PC1 (25.73%)
7
6
5
4
3
2
1
0
-1
-2
-3
-4
-2
0
2
4
6
8
10
c55z2
c55z3
c55z3
c57z2
c56z2
c56z1
c58z3
c56z3
c57z1
c57z3
c58z2
c58z5
Groups
WT/WT
WT/rif2Δ
WT/WT_long1
WT/WT_long2

Figure S1
