# Supplementary material for: Inherited long telomeres induce a genome-wide transcriptional response in budding yeast
Source: Genetics. 2026 Jul 3;234(2):iyag174. doi: 10.1093/genetics/iyag174 (PMC13643770; doi:10.1093/genetics/iyag174)
Supplement: Supplementary file 1 [file iyag174_supplementary_data.zip › Figure_S2_GENETICS-2026-309600.pdf]

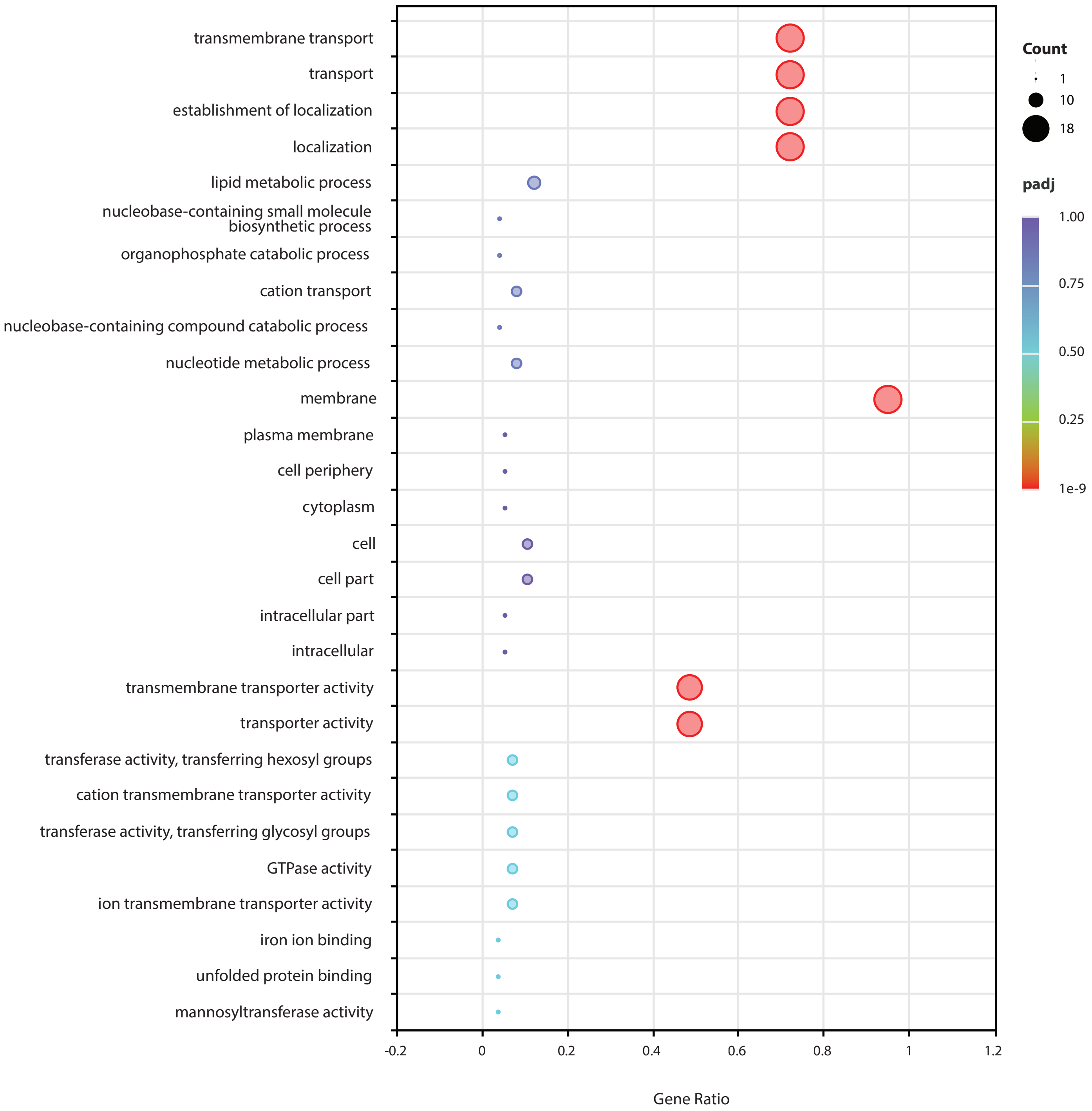

transmembrane transport
transport
establishment of localization
localization
lipid metabolic process
nucleobase-containing small molecule biosynthetic process
organophosphate catabolic process
cation transport
nucleobase-containing compound catabolic process
nucleotide metabolic process
membrane
plasma membrane
cell periphery
cytoplasm
cell
cell part
intracellular part
intracellular
transmembrane transporter activity
transporter activity
transferase activity, transferring hexosyl groups
cation transmembrane transporter activity
transferase activity, transferring glycosyl groups
GTPase activity
ion transmembrane transporter activity
iron ion binding
unfolded protein binding
mannosyltransferase activity
-0.2
0
0.2
0.4
0.6
0.8
1
1.2
Gene Ratio
Count
1
10
18
padj
1.00
0.75
0.50
0.25
1e-9

Figure S2
