# Supplementary material for: Inherited long telomeres induce a genome-wide transcriptional response in budding yeast
Source: Genetics. 2026 Jul 3;234(2):iyag174. doi: 10.1093/genetics/iyag174 (PMC13643770; doi:10.1093/genetics/iyag174)
Supplement: Supplementary file 1 [file iyag174_supplementary_data.zip › File_S1_GENETICS-2026-309600.pdf]

File S1: Lists of differentially expressed genes across the three pairwise comparisons (outlier c56z1 excluded)

1) *WT/WT_long1* vs *WT/WT*

Gene ID	Fold change	Adjusted p–value	Gene standard name and function
YDL223C	1.89	1.57E-12	*HBT1 – Protein Hbt1*
YDL222C	1.60	2.08E-18	*FMP45* – SUR7 family protein Fmp45
YDL210W	1.54	6.80E-06	*UGA4* – GABA–specific permease
YHR096C	1.46	1.90E-07	*HXT5* – Probable glucose transporter Hxt5
YNL160W	1.46	1.87E-13	*YGP1* – Protein Ygp1
YCR061W	1.45	0.0027	*YCV1* – Uncharacterized membrane protein
YPL223C	1.43	5.89E-05	*GRE1* – Protein Gre1
YNL195C	1.42	6.92E-05	*YNT5* – Uncharacterized protein
YDL214C	1.41	0.0002	*PRR2* – Serine/threonine-protein kinase Prr2
YOL084W	1.40	0.0002	*PHM7* – Phosphate metabolism protein 7
YHL016C	1.39	0.0003	*DUR3* – Urea active transporter
YBR117C	1.38	0.0015	*TKT2* – Transketolase 2
YBL075C	1.36	0.0011	*HSP73* – Heat shock protein Ssa3
YBR054W	1.36	1.82E-08	*YRO2* – Protein Yro2
YML123C	1.36	0.0006	*PHO84* – Inorganic phosphate transporter Pho84
novel.28	1.35	0.0008	*YB21B* – Transposon Ty2–B Gag–Pol polyprotein

Table continued on next page.

**Gene ID**	**Fold change**	**Adjusted p–value**	**Gene standard name and function**
YMR306W	1.35	0.0038	*FKS3* – 1,3–beta–glucan synthase component Fks3
YML128C	1.34	3.53E-07	*MSC1* – Meiotic sister chromatid recombination protein 1
YNL142W	1.33	0.0002	*MEP2* – Ammonium transporter Mep2
YOR328W	1.33	0.0002	*PDR10* – ATP–dependent permease Pdr10
YPR026W	1.33	0.0016	*ATH1* – Periplasmic acid trehalase Atc1
YNL194C	1.33	1.53E-06	*YNT4* – Uncharacterized plasma membrane protein
YDR406W	1.33	0.0235	*PDR15* – ATP–dependent permease Pdr15
YLR413W	1.33	0.0033	*YL413* – Cell membrane protein
YLR450W	1.33	0.0110	*HMDH2* – 3–hydroxy–3–methylglutaryl–coenzyme A reductase 2
YDR205W	1.33	0.0006	*MSC2* – Probable zinc transporter Msc2
YNL014W	1.32	0.0191	*EF3B* – Elongation factor 3B
YGL008C	1.31	0.0270	*PMA1* – Plasma membrane ATPase 1
YJR094C	1.31	0.0030	*IME1* – Meiosis–inducing protein 1
YGL055W	1.31	5.23E-05	*ACO1* – Acyl–CoA desaturase 1
YOL119C	1.31	0.0001	*MCH4* – Probable transporter Mch4
YKL187C	1.30	0.0030	*YKS7* – Uncharacterized protein

Table continued on next page.

Gene ID	Fold change	Adjusted p–value	Gene standard name and function
YMR266W	1.30	0.0047	*RSN1* – Uncharacterized protein Rsn1
YBR235W	1.29	0.0028	*VHC1* – Vacuolar cation–chloride cotransporter 1
YJL045W	1.29	0.0031	*SDHX* – Succinate dehydrogenase [ubiquinone] flavoprotein subunit 2, mitochondrial
YGR032W	1.29	0.0466	*FKS2* – 1,3–beta–glucan synthase component Gsc2
YJR106W	1.29	0.0063	*ECM27* – Protein Ecm27
YER150W	1.29	0.0255	*SPI1* – Uncharacterized cell wall protein Spi1
YJL172W	1.29	0.0103	*CBPS* – Carboxypeptidase S
YPL006W	1.28	0.0376	*NPC1* – NPC intracellular sterol transporter 1–related protein 1
YDR420W	1.28	0.0253	*HKR1* – Signalling mucin Hkr1
YCR021C	1.28	0.0035	*HSP30* – 30 kDa heat shock protein
YIL030C	1.28	0.0030	*DOA10* – ERAD–associated E3 ubiquitin–protein ligase Doa10
YGR143W	1.28	0.0103	*SKN1* – Beta–glucan synthesis–associated protein Skn1
YGR281W	1.28	0.0351	*YOR1* – Oligomycin resistance ATP–dependent permease Yor1
YCR032W	1.27	0.0188	*BPH1* – Beige protein homolog 1
novel.119	1.27	0.0025	*TPO3* – Polyamine transporter 3
YKL050C	1.27	0.0431	*YKF0* – Uncharacterized protein
YDL204W	1.27	2.87E-05	*RTN2* – Reticulon–like protein 2

Table continued on next page.

Gene ID	Fold change	Adjusted p–value	Gene standard name and function
YCR068W	1.27	0.0003	*ATG15* – Putative lipase Atg15
YKL034W	1.27	0.0185	*TUL1* – Transmembrane E3 ubiquitin–protein ligase 1
YPL275W	1.27	0.0286	*FDH2* – Truncated formate dehydrogenase 2
YML075C	1.26	0.0218	*HMDH1* – 3–hydroxy–3–methylglutaryl–coenzyme A reductase 1
novel.22	1.26	0.0305	*YD22B* – Transposon Ty2–DR2 Gag–Pol polyprotein
YMR034C	1.26	0.0011	*RCH1* – Solute carrier Rch1
YLL015W	1.26	0.0418	*BPT1* – Bile pigment transporter 1
YIL119C	1.26	0.0230	*RPI1* – Negative RAS protein regulator protein
YBR072W	1.26	0.0218	*HSP26* – Heat shock protein 26
YPL137C	1.26	0.0384	*GIP3* – GLC7–interacting protein 3
novel.371	1.26	0.0286	*PPA5* – Repressible acid phosphatase
YDL199C	1.26	0.0351	*YD199* – Putative metabolite transport protein
YLL055W	1.25	0.0093	*YCT1* – High affinity cysteine transporter
YDR135C	1.25	0.0244	*YCFI* – Metal resistance protein Ycf1
YPL265W	1.25	8.02E-05	*DIP5* – Dicarboxylic amino acid permease
YKL198C	1.25	0.0490	*PTK1* – Serine/threonine-protein kinase Ptk1/Stk1

Table continued on next page.

Gene ID	Fold change	Adjusted p–value	Gene standard name and function
YHR202W	1.24	0.0009	*YH02* – Uncharacterized protein
YOL101C	1.24	0.0215	*IZH4* – ADIPOR–like receptor Izh4
YGR260W	1.24	0.0028	*TNA1* – High–affinity nicotinic acid transporter
YLR152C	1.24	0.0114	*YL152* – Uncharacterized transporter
YHR056C	1.24	0.0253	*RSC30* – Chromatin structure-remodelling complex protein Rsc30
YGL027C	1.24	0.0293	*CWH41* – Mannosyl–oligosaccharide glucosidase
YLR312C	1.24	0.0038	*ATG39* – Autophagy–related protein 39
YBL004W	1.23	0.0253	*UTP20* – U3 small nucleolar RNA–associated protein 20
YNL237W	1.23	0.0093	*YTP1* – Protein Ytp1
YJL005W	1.23	0.0404	*CYAA* – Adenylate cyclase
YCR017C	1.23	0.0030	*CWH43* – Protein Cwh43
YHR080C	1.23	0.0353	*LAM4* – Membrane-anchored lipid–binding protein Lam4
YOR391C	1.23	0.0253	*HSP33* – Probable glutathione-independent glyoxalase Hsp33
YGL006W	1.23	0.0229	*ATC2* – Calcium–transporting ATPase 2
YLR371W	1.23	0.0253	*ROM2* – Rho1 GDP–GTP exchange protein 2
YBR241C	1.23	0.0011	*YB91* – Probable metabolite transport protein

Table continued on next page.

Gene ID	Fold change	Adjusted p–value	Gene standard name and function
YGR121C	1.22	0.0186	*MEP1* – Ammonium transporter Mep1
YLR207W	1.22	0.0075	*HRD3* – ERAD–associated E3 ubiquitin–protein ligase component Hrd3
YDL237W	1.21	0.0200	*AIM6* – Altered inheritance of mitochondria protein 6
YJL163C	1.21	0.0212	*YJQ3* – Uncharacterized membrane protein
YKL217W	1.21	0.0036	*JEN1* – Carboxylic acid transporter protein homolog
YIL162W	1.21	0.0214	*INV2* – Invertase 2
YLR299W	1.21	0.0186	*ECM38* – Glutathione hydrolase proenzyme
YDR307W	1.20	0.0230	*PMT7* – Probable dolichyl–phosphate-mannose-–protein mannosyltransferase 7
YNR067C	1.20	0.0466	*ENG1* – Glucan endo–1,3–beta–D–glucosidase 1
YHR028C	1.20	0.0478	*DAP2* – Dipeptidyl aminopeptidase B
YBR067C	1.20	0.0016	*TIP1* – Temperature shock–inducible protein 1
YKL140W	1.20	0.0335	*TGL1* – Sterol esterase Tgl1
YHR039C	1.20	0.0380	*MSC7* – Putative aldehyde dehydrogenase-like protein
YGL186C	1.20	0.0218	*TPN1* – Vitamin B6 transporter Tpn1
YDL054C	1.20	0.0200	*MCH1* – Probable transporter Mch1
novel.23	1.19	0.0302	*TY1AB* – Transposon TyH3 Gag–Pol polyprotein

Table continued on next page.

Gene ID	Fold change	Adjusted p–value	Gene standard name and function
YKL008C	1.19	0.0253	*LAC1* – Sphingosine N–acyltransferase Lac1
YNL115C	1.18	0.0208	*YNL5* – Uncharacterized vacuolar membrane protein
YNL268W	1.18	0.0218	*LYP1* – Lysine-specific permease
YBR286W	1.18	0.0095	*APE3* – Aminopeptidase Y
YOR002W	1.18	0.0466	*ALG6* – Dolichyl pyrophosphate Man9GlcNAc2 alpha–1,3–glucosyltransferase
YOL013C	1.18	0.0253	*HRD1* – ERAD–associated E3 ubiquitin–protein ligase Hrd1
YGR060W	1.18	0.0186	*ERG25* – C–4 methylsterol oxidase Erg25
YDL128W	1.18	0.0439	*VCX1* – Vacuolar calcium ion transporter
YDR492W	1.18	0.0235	*IZH1* – ADIPOR–like receptor Izh1
novel.318	1.17	0.0230	*ATR* – Serine/threonine-protein kinase Mec1
YDR483W	1.17	0.0381	*KRE2* – Glycolipid 2–alpha–mannosyltransferase
YIL005W	1.17	0.0490	*EPS1* – ER–retained PMA1–suppressing protein 1
YDR536W	1.17	0.0428	*STL1* – Sugar transporter Stl1
YHR132C	1.15	0.0466	*ECM14* – Inactive metallocarboxypeptidase Ecm14
YFL045C	0.85	0.0428	*PMM* – Phosphomannomutase
YJR133W	0.85	0.0479	*XPT1* – Xanthine phosphoribosyltransferase 1
YGR165W	0.85	0.0466	*RT35* – Small ribosomal subunit protein mS45

Table continued on next page.

Gene ID	Fold change	Adjusted p–value	Gene standard name and function
YML124C	0.85	0.0398	*TBA3* – Tubulin alpha–3 chain
YKL085W	0.85	0.0230	*MDHM* – Malate dehydrogenase, mitochondrial
YBR010W	0.85	0.0490	*H3* – Histone H3
YOR327C	0.84	0.0469	*SNC2* – Synaptobrevin homolog 2
YNL284C	0.84	0.0466	*RM10* – Large ribosomal subunit protein uL15m
YDR316W	0.84	0.0230	*OMS1* – Methyltransferase Oms1, mitochondrial
YFL016C	0.84	0.0046	*MDJ1* – DnaJ homolog 1, mitochondrial
YMR024W	0.84	0.0255	*RM03* – Large ribosomal subunit protein mL44
YKR006C	0.84	0.0274	*RM13* – Large ribosomal subunit protein mL50
YGR082W	0.84	0.0281	*TOM20* – Mitochondrial import receptor subunit Tom20
YPL098C	0.84	0.0476	*MGR2* – Protein Mgr2
YJR101W	0.83	0.0281	*RT26* – Small ribosomal subunit protein mS42
YGL068W	0.83	0.0466	*MNP1* – Large ribosomal subunit protein bL12m
YOL053W	0.83	0.0261	*AIM39* – Altered inheritance of mitochondria protein 39, mitochondrial
YNL067W	0.83	0.0381	*RL9B* – Large ribosomal subunit protein uL6B
YNL073W	0.83	0.0258	*SYKM* – Lysine-–tRNA ligase, mitochondrial

Table continued on next page.

Gene ID	Fold change	Adjusted p–value	Gene standard name and function
YLR069C	0.83	0.0171	*EFGM* – Elongation factor G, mitochondrial
YDR337W	0.82	0.0229	*RT28* – Small ribosomal subunit protein uS15m
YDR487C	0.82	0.0214	*RIB3* – 3,4–dihydroxy–2–butanone 4–phosphate synthase
YPL256C	0.82	0.0398	*CG12* – G1/S–specific cyclin Cln2
YKL195W	0.82	0.0008	*MIA40* – Mitochondrial intermembrane space import and assembly protein 40
YKR013W	0.82	0.0255	*PRY2* – Protein Pry2
YLR168C	0.82	0.0075	*UPS2* – Protein Ups2, mitochondrial
YJL158C	0.82	0.0116	*CIS3* – Cell wall mannoprotein Cis3
YKL216W	0.81	0.0018	*PYRD* – Dihydroorotate dehydrogenase (fumarate)
YDL235C	0.81	0.0466	*YPD1* – Phosphorelay intermediate protein Ypd1
YBL038W	0.81	0.0228	*RM16* – Large ribosomal subunit protein uL16m
YIL070C	0.81	0.0093	*MAM33* – Mitochondrial acidic protein Mam33
YLR204W	0.81	0.0132	*COX24* – Small ribosomal subunit protein mS38
YMR193W	0.81	0.0068	*RM24* – Large ribosomal subunit protein bL28m
YNR022C	0.81	0.0286	*RM50* – Large ribosomal subunit protein bL9m
YOR187W	0.80	5.23E-05	*EFTU* – Elongation factor Tu, mitochondrial

Table continued on next page.

Gene ID	Fold change	Adjusted p–value	Gene standard name and function
YPR158W	0.80	0.0116	*CUR1* – Curing of [URE3] protein 1
YBL090W	0.80	0.0404	*RT21* – Small ribosomal subunit protein bS21m
YNL289W	0.80	0.0015	*PCL1* – Pho85 cyclin–1
YMR157C	0.80	0.0232	*AIM36* – Altered inheritance of mitochondria protein 36, mitochondrial
YJR048W	0.79	0.0018	*CYC1* – Cytochrome c isoform 1
YNL300W	0.79	0.0150	*TOS6* – Protein TOS6
YFR001W	0.79	0.0218	*LOC1* – 60S ribosomal subunit assembly/export protein Loc1
YBR009C	0.78	0.0006	*H4* – Histone H4
YNR049C	0.77	0.0234	*MSO1* – Protein Mso1
YBL003C	0.76	0.0019	*H2A2* – Histone H2A.2
YHR059W	0.75	0.0028	*FYV4* – Small ribosomal subunit protein mS41
YNL030W	0.74	4.85E-05	*H4* – Histone H4
novel.337	0.74	0.0220	*COX17* – Cytochrome c oxidase copper chaperone
YBL002W	0.67	3.94E-05	*H2B2* – Histone H2B.2

2) *WT/WT_long2* vs *WT/WT*

Gene ID	Fold change	Adjusted p–value	Gene standard name and function
YOR338W	2.09	0.0444	*YO338* – SWIRM domain–containing protein
YDL223C	1.92	0.0238	*HBT1* – Protein Hbt1
novel.37	1.87	0.0301	*SSF1* – Ribosome biogenesis protein Ssf1
YPL036W	1.76	0.0050	*PMA2* – Plasma membrane ATPase 2
novel.321	1.75	0.0005	*DBP2* – ATP–dependent RNA helicase Dbp2
YBR105C	1.69	0.0013	*GID4* – Vacuolar import and degradation protein 24
novel.449	1.65	0.0013	–
YBR296C	1.60	0.0011	*PHO89* – Phosphate permease Pho89
YBR117C	1.60	0.0036	*TKT2* – Transketolase 2
YNL160W	1.58	1.35E-08	*YGP1* – Protein Ygp1
YBR054W	1.57	0.0005	*YRO2* – Protein Yro2
YER187W	1.56	0.0422	*YE17* – Uncharacterized protein
YGR079W	1.54	0.0037	*YG2G* – Putative uncharacterized protein
YHR096C	1.54	0.0005	*HXT5* – Probable glucose transporter Hxt5
YOR359W	1.53	0.0224	*VTS1* – RNA–binding protein Vts1
YKR075C	1.51	0.0011	*YK55* – Uncharacterized protein
YOR107W	1.50	0.0103	*RGS2* – Regulator of G–protein signaling 2

Table continued on next page.

Gene ID	Fold change	Adjusted p–value	Gene standard name and function
YER127W	1.49	0.0316	*LCP5* – U3 small nucleolar ribonucleoprotein protein Lcp5
YGL008C	1.46	0.0027	*PMA1* – Plasma membrane ATPase 1
YMR095C	1.46	0.0036	*SNO1* – Pyridoxal 5'–phosphate synthase subunit Sno1
YML116W	1.46	0.0035	*ATR1* – Aminotriazole resistance protein
YBR238C	1.45	0.0172	*RMD9L* – RNA–binding protein Rmd9-like, mitochondrial
YDL048C	1.44	0.0008	*STP4* – Zinc finger protein Stp4
YLR413W	1.43	0.0066	*YL413* – Cell membrane protein
YMR306W	1.42	0.0027	*FKS3* – 1,3–beta–glucan synthase component Fks3
YDL210W	1.42	0.0005	*UGA4* – GABA–specific permease
YOR360C	1.42	0.0046	*PDE2* – 3',5'–cyclic–nucleotide phosphodiesterase 2
novel.119	1.42	0.0005	*TPO3* – Polyamine transporter 3
YGL186C	1.41	0.0005	*TPN1* – Vitamin B6 transporter Tpn1
YIL053W	1.41	0.0236	*GPP1* – Glycerol–1–phosphate phosphohydrolase 1
YOL084W	1.40	0.0074	*PHM7* – Phosphate metabolism protein 7
YPL026C	1.40	0.0054	*SKS1* – Serine/threoninE-protein kinase Sks1
YER150W	1.40	0.0207	*SPI1* – Uncharacterized cell wall protein Spi1
YNL234W	1.39	0.0441	*YNX4* – Uncharacterized globin–like protein

Table continued on next page.

Gene ID	Fold change	Adjusted p–value	Gene standard name and function
YCR061W	1.39	0.0294	*YCV1* – Uncharacterized membrane protein
YGL028C	1.39	0.0053	*SCW11* – Probable family 17 glucosidase Scw11
YDR465C	1.38	0.0310	*RMT2* – Protein arginine N–methyltransferase 2
YML123C	1.38	0.0005	*PHO84* – Inorganic phosphate transporter Pho84
YDR277C	1.38	0.0036	*MTH1* – Protein Mth1
YJL116C	1.37	0.0417	*NCA3* – Beta–glucosidasE-like protein Nca3, mitochondrial
YOL119C	1.37	0.0009	*MCH4* – Probable transporter Mch4
YDR406W	1.37	0.0238	*PDR15* – ATP–dependent permease Pdr15
YGL055W	1.36	0.0010	*ACO1* – Acyl–CoA desaturase 1
YIL162W	1.36	0.0207	*INV2* – Invertase 2
YBL075C	1.36	0.0316	*HSP73* – Heat shock protein Ssa3
novel.83	1.35	0.0238	*TPO3* – Polyamine transporter 3
YHR170W	1.34	0.0301	*NMD3* – 60S ribosomal export protein Nmd3
YCR021C	1.34	0.0303	*HSP30* – 30 kDa heat shock protein
YNL014W	1.34	0.0160	*EF3B* – Elongation factor 3B
YMR085W	1.34	0.0385	*YM084* – Putative glutamine-fructose-6-phosphate aminotransferase [isomerizing]
YNL195C	1.33	0.0105	*YNT5* – Uncharacterized protein

Table continued on next page.

Gene ID	Fold change	Adjusted p–value	Gene standard name and function
YDL063C	1.33	0.0257	*SYO1* – Synchronized import protein 1
YGR052W	1.33	0.0236	*FMP48* – Probable serine/threonine-protein kinase Fmp48
YPL043W	1.33	0.0297	*NOP4* – Nucleolar protein 4
YDR527W	1.33	0.0320	*RBA50* – RNA polymerase II–associated protein Rba50
YKL187C	1.33	0.0238	*YKS7* – Uncharacterized protein
YBR072W	1.32	0.0497	*HSP26* – Heat shock protein 26
YOR049C	1.32	0.0269	*RSB1* – Sphingoid long–chain base transporter Rsb1
YDL204W	1.32	0.0224	*RTN2* – Reticulon–like protein 2
YNR067C	1.32	0.0027	*ENG1* – Glucan endo–1,3–beta–D–glucosidase 1
YGR191W	1.32	0.0025	*HIP1* – Histidine permease
YKL034W	1.32	0.0139	*TUL1* – Transmembrane E3 ubiquitin–protein ligase 1
YML128C	1.32	0.0069	*MSC1* – Meiotic sister chromatid recombination protein 1
YDL214C	1.31	0.0140	*PRR2* – Serine/threoninE-protein kinase Prr2
YNL124W	1.31	0.0341	*NAF1* – H/ACA ribonucleoprotein complex non–core subunit Naf1
YIL030C	1.31	0.0074	*DOA10* – ERAD–associated E3 ubiquitin–protein ligase Doa10
YDR075W	1.31	0.0497	*PP4C* – Serine/threonine-protein phosphatase 4 catalytic subunit
YJL033W	1.31	0.0196	*DBP4* – ATP–dependent RNA helicase Dbp4

Table continued on next page.

Gene ID	Fold change	Adjusted p–value	Gene standard name and function
novel.28	1.31	0.0257	*YB21B* – Transposon Ty2–B Gag–Pol polyprotein
YDR205W	1.31	0.0115	*MSC2* – Probable zinc transporter Msc2
YKR098C	1.30	0.0070	*UBP11* – Ubiquitin carboxyl–terminal hydrolase 11
YHL016C	1.30	0.0220	*DUR3* – Urea active transporter
YLR450W	1.30	0.0409	*HMDH2* – 3–hydroxy–3–methylglutaryl–coenzyme A reductase 2
YML118W	1.30	0.0294	*NGL3* – Probable RNA exonuclease Ngl3
YCR068W	1.30	0.0053	*ATG15* – Putative lipase Atg15
YHR143W	1.30	0.0268	*DSE2* – Protein Dse2
YER052C	1.30	0.0020	*AK* – Aspartokinase
YGR143W	1.30	0.0150	*SKN1* – Beta–glucan synthesis–associated protein Skn1
YPL126W	1.30	0.0165	*UTP17* – NET1–associated nuclear protein 1
YHR052W	1.30	0.0376	*CIC1* – ProteasomE-interacting protein Cic1
YDR247W	1.30	0.0207	*VHS1* – Serine/threoninE-protein kinase Vhs1
YJL172W	1.29	0.0294	*CBPS* – Carboxypeptidase S
YMR189W	1.29	0.0320	*GCSP* – Glycine dehydrogenase (decarboxylating), mitochondrial
YMR096W	1.29	0.0446	*SNZ1* – Pyridoxal 5'–phosphate synthase subunit Snz1
YOR337W	1.29	0.0160	*TEA1* – TY1 enhancer activator

Table continued on next page.

Gene ID	Fold change	Adjusted p–value	Gene standard name and function
YBR235W	1.29	0.0143	*VHC1* – Vacuolar cation–chloride cotransporter 1
YMR266W	1.29	0.0309	*RSN1* – Uncharacterized protein Rsn1
YLL010C	1.29	0.0365	*PSR1* – Phosphatase Psr1
YDR496C	1.28	0.0231	*PUF6* – Pumilio homology domain family member 6
YDR033W	1.28	0.0241	*MRH1* – Protein Mrh1
YPL092W	1.28	0.0497	*SSU1* – Sulfite efflux pump Ssu1
YNL190W	1.28	0.0034	Uncharacterized protein
YMR319C	1.28	0.0039	*FET4* – Low–affinity Fe(2+) transport protein
YBR114W	1.28	0.0168	*RAD16* – DNA repair protein Rad16
YOR204W	1.28	0.0353	*DED1* – ATP–dependent RNA helicase Ded1
YPL137C	1.27	0.0148	*GIP3* – Glc7–interacting protein 3
YGR197C	1.27	0.0207	*SNG1* – Nitrosoguanidine resistance protein Sng1
YBR067C	1.27	0.0025	*TIP1* – Temperature shock–inducible protein 1
YDR135C	1.27	0.0287	*YCFI* – Metal resistance protein Ycf1
YHR150W	1.27	0.0376	*PEX28* – Peroxisomal membrane protein Pex28
YMR034C	1.27	0.0051	*RCH1* – Solute carrier Rch1
YIL130W	1.27	0.0074	*ASG1* – Activator of stress genes 1

Table continued on next page.

Gene ID	Fold change	Adjusted p–value	Gene standard name and function
YHR202W	1.27	0.0089	*YH02* – Uncharacterized protein
YIL056W	1.26	0.0316	*VHR1* – Transcription factor Vhr1
YGR245C	1.26	0.0392	*SDA1* – Protein Sda1
YMR088C	1.26	0.0429	*VBA1* – Vacuolar basic amino acid transporter 1
YER056C	1.26	0.0084	*FCY2* – PurinE-cytosine permease Fcy2
YOR391C	1.26	0.0290	*HSP33* – Probable glutathionE-independent glyoxalase Hsp33
YNL237W	1.26	0.0143	*YTP1* – Protein Ytp1
YOR328W	1.26	0.0497	*PDR10* – ATP–dependent permease Pdr10
YMR031C	1.26	0.0159	*EIS1* – Eisosome protein 1
YML075C	1.26	0.0417	*HMDH1* – 3–hydroxy–3–methylglutaryl–coenzyme A reductase 1
YCR017C	1.26	0.0084	*CWH43* – Protein Cwh43
YJL163C	1.26	0.0234	*YJQ3* – Uncharacterized membrane protein
YCL030C	1.25	0.0438	*HIS2* – Histidine biosynthesis trifunctional protein
YGL006W	1.25	0.0316	*ATC2* – Calcium–transporting ATPase 2
YBR074W	1.25	0.0316	*PFF1* – Vacuolar membrane protease
YDL138W	1.25	0.0129	*RGT2* – High glucose sensor Rgt2
YOR002W	1.25	0.0159	*ALG6* – Dolichyl pyrophosphate Man9GlcNAc2 alpha–1,3–glucosyltransferase

Table continued on next page.

Gene ID	Fold change	Adjusted p–value	Gene standard name and function
YJL078C	1.25	0.0365	*PRY3* – Cell wall protein Pry3
YLR299W	1.24	0.0236	*ECM38* – Glutathione hydrolase proenzyme
YOR303W	1.24	0.0070	*CARA* – Carbamoyl phosphate synthase argininE-specific small chain
YDR536W	1.24	0.0205	*STL1* – Sugar transporter Stl1
YGR260W	1.24	0.0107	*TNA1* – High–affinity nicotinic acid transporter
YLL055W	1.24	0.0278	*YCT1* – High affinity cysteine transporter
YML130C	1.23	0.0236	*ERO1* – Endoplasmic oxidoreductin–1
YJR109C	1.23	0.0497	*CARB* – Carbamoyl phosphate synthase argininE-specific large chain
YDR128W	1.23	0.0376	*WDR59* – Maintenance of telomere capping protein 5
YNL142W	1.23	0.0316	*MEP2* – Ammonium transporter Mep2
YHR028C	1.23	0.0497	*DAP2* – Dipeptidyl aminopeptidase B
YDL237W	1.23	0.0353	*AIM6 – Altered inheritance of mitochondria protein 6*
YCR098C	1.23	0.0360	*GIT1* – Glycerophosphoinositol transporter 1
YDL054C	1.22	0.0228	*MCH1* – Probable transporter Mch1
YMR297W	1.22	0.0220	*CBPY* – Carboxypeptidase Y
YMR243C	1.22	0.0224	*ZRC1* – Vacuolar zinc transporter Zrc1

Table continued on next page.

Gene ID	Fold change	Adjusted p–value	Gene standard name and function
YDR422C	1.22	0.0444	*SIP1* – SNF1 protein kinase subunit beta–1
YNL280C	1.22	0.0316	*ERG24* – Delta(14)–sterol reductase Erg24
YPL265W	1.22	0.0074	*DIP5* – Dicarboxylic amino acid permease
YOR256C	1.21	0.0487	*TRE2* – Putative zinc metalloprotease Tre2
YDR492W	1.21	0.0385	*IZH1* – ADIPOR–like receptor Izh1
YDR483W	1.21	0.0264	*KRE2* – Glycolipid 2–alpha–mannosyltransferase
YLR207W	1.21	0.0376	*HRD3* – ERAD–associated E3 ubiquitin–protein ligase component Hrd3
YBR286W	1.21	0.0129	*APE3* – Aminopeptidase Y
YML006C	1.21	0.0497	*GIS4* – Protein Gis4
YNL268W	1.20	0.0279	*LYP1* – Lysine-specific permease
YPR083W	1.20	0.0399	*MDM36* – Mitochondrial distribution and morphology protein 36
YLR248W	1.19	0.0495	*RCK2* – Serine/threoninE-protein kinase Rck2
YGR060W	1.18	0.0376	*ERG25* – C–4 methylsterol oxidase Erg25
YFL016C	0.86	0.0448	*MDJ1* – DnaJ homolog 1, mitochondrial
YOR187W	0.85	0.0297	*EFTU* – Elongation factor Tu, mitochondrial
YKL195W	0.85	0.0238	*MIA40* – Mitochondrial intermembrane space import and assembly protein 40

Table continued on next page.

Gene ID	Fold change	Adjusted p–value	Gene standard name and function
YHR174W	0.84	0.0484	*ENO1* – Enolase 1
YLR069C	0.84	0.0429	*EFGM* – Elongation factor G, mitochondrial
YDR316W	0.84	0.0316	*OMS1* – Methyltransferase Oms1, mitochondrial
YOR327C	0.83	0.0407	*SNC2* – Synaptobrevin homolog 2
YKL216W	0.83	0.0429	*PYRD* – Dihydroorotate dehydrogenase (fumarate)
YLR314C	0.83	0.0251	*CDC3* – Cell division control protein 3
YPR101W	0.82	0.0348	*SN309* – PrE-mRNA–splicing factor Snt309
YGL068W	0.82	0.0367	*MNP1* – Large ribosomal subunit protein bL12m
YIL070C	0.82	0.0236	*MAM33* – Mitochondrial acidic protein Mam33
YCL034W	0.82	0.0376	*LSB5* – LAS seventeen–binding protein 5
YGR189C	0.82	0.0297	*CRH1* – Probable glycosidase Crh1
YNL058C	0.82	0.0124	*YNF8* – Vacuolar membrane protein
YNL056W	0.81	0.0417	*OCA2* – TyrosinE-protein phosphatasE-like protein Oca2
YOR195W	0.81	0.0219	*SLK19* – Kinetochore protein Slk19
YJR048W	0.81	0.0166	*CYC1* – Cytochrome c isoform 1
YHR137W	0.80	0.0430	*ARO9* – Aromatic amino acid aminotransferase 2

Table continued on next page.

**Gene ID**	**Fold change**	**Adjusted p–value**	**Gene standard name and function**
YJR076C	0.80	0.0221	*CDC11* – Cell division control protein 11
YOL012C	0.80	0.0497	*H2AZ* – Histone H2A.Z
YKL096W	0.80	0.0196	*CWP1* – Cell wall protein Cwp1
YLR168C	0.80	0.0074	*UPS2* – Protein Ups2, mitochondrial
YHR209W	0.80	0.0148	*CRG1* – Probable S–adenosylmethionine-dependent methyltransferase Crg1
YPR158W	0.80	0.0236	*CUR1* – Curing of [URE3] protein 1
YML102W	0.80	0.0297	*CAF1B* – Chromatin assembly factor 1 subunit B
YFL045C	0.79	0.0448	*PMM* – Phosphomannomutase
YBR010W	0.79	0.0224	*H3* – Histone H3
YHR059W	0.79	0.0357	*FYV4* – Small ribosomal subunit protein mS41
YLR151C	0.79	0.0105	*PCD1* – Peroxisomal coenzyme A diphosphatase 1, peroxisomal
YDR315C	0.78	0.0316	*IPK1* – Inositol–pentakisphosphate 2–kinase
YFL037W	0.78	0.0251	*TBB* – Tubulin beta chain
YDL157C	0.78	0.0497	*YD157* – Uncharacterized protein, mitochondrial
YGR040W	0.77	0.0497	*KSS1* – Mitogen–activated protein kinase Kss1
YFR001W	0.77	0.0160	*LOC1* – 60S ribosomal subunit assembly/export protein Loc1
YIL004C	0.77	0.0165	*BET1* – Protein transport protein Bet1

Table continued on next page.

Gene ID	Fold change	Adjusted p–value	Gene standard name and function
YER030W	0.76	0.0446	*CHZ1* – Histone H2A.Z–specific chaperone Chz1
YDL101C	0.76	0.0020	*DUN1* – DNA damage response protein kinase Dun1
YJL153C	0.76	0.0381	*INO1* – Inositol–3–phosphate synthase
YDR013W	0.76	0.0367	*PSF1* – DNA replication complex GINS protein Psf1
YNL090W	0.75	0.0113	*RHO2* – GTP–binding protein Rho2
YLR200W	0.75	0.0224	*PFD6* – Prefoldin subunit 6
YNL126W	0.75	0.0444	*SPC98* – Spindle pole body component Spc98
YOR373W	0.74	0.0251	*NUD1* – Protein NUD1
YLR353W	0.74	0.0196	*BUD8* – Bud site selection protein 8
YOR025W	0.74	0.0171	*HST3* – NAD–dependent histone deacetylase Hst3
novel.337	0.74	0.0251	*COX17* – Cytochrome c oxidase copper chaperone
YBR088C	0.74	0.0224	*PCNA* – Proliferating cell nuclear antigen
YJR107C_A	0.73	0.0353	*YJ107* – Uncharacterized protein
YCR003W	0.72	0.0234	*RM32* – Large ribosomal subunit protein bL32m
YDL219W	0.72	0.0497	*DTD* – D–aminoacyl–tRNA deacylase
YOR247W	0.72	0.0086	*YO248* – Putative uncharacterized protein
novel.406	0.72	0.0122	*STF1* – F(1)–ATPase inhibitor Stf1, mitochondrial

Table continued on next page.

**Gene ID**	**Fold change**	**Adjusted p–value**	**Gene standard name and function**
YDR451C	0.71	0.0220	*YHP1* – Homeobox protein Yhp1
YDR224C	0.70	0.0078	*H2B1* – Histone H2B.1
YBR009C	0.69	0.0027	*H4 – Histone H4*
YGR096W	0.67	0.0236	*TPC1* – Mitochondrial thiamine pyrophosphate carrier 1
novel.260	0.65	0.0429	*RL6A* – Large ribosomal subunit protein eL6A
YPR078C	0.64	0.0207	*YP078* – Uncharacterized protein
YNL030W	0.63	0.0007	*H4* – Histone H4
YDR222W	0.62	0.0367	*YDR22* – SVF1–like protein
YEL067C	0.60	0.0438	*YEH7* – Uncharacterized protein
YDR209C	0.59	0.0234	*YD209* – Uncharacterized protein
novel.360	0.46	0.0487	–

3) *WT/rif2Δ* vs *WT/WT*

Gene ID	Fold change	Adjusted p–value	Gene standard name and function
novel.1	8.04	8.15E-12	*REP1* – Partitioning protein Rep1
novel.4	4.55	7.06E-08	*FLP* – Site-specific recombinase Flp
YGL028C	2.01	4.40E-11	*SCW11* – Probable family 17 glucosidase Scw11
YER124C	1.82	1.58E-10	*DSE1* – Protein Dse1
YHR143W	1.79	2.05E-08	*DSE2* – Protein Dse2
YHR096C	1.78	2.59E-12	*HXT5* – Probable glucose transporter Hxt5
YDL222C	1.67	2.00E-13	*FMP45* – SUR7 family protein Fmp45
YML123C	1.64	8.60E-09	*PHO84* – Inorganic phosphate transporter Pho84
novel.371	1.64	2.05E-08	*PPA5* – Repressible acid phosphatase
YJL078C	1.63	4.86E-06	*PRY3* – Cell wall protein Pry3
YDL223C	1.62	1.29E-06	*HBT1* – Protein Hbt1
YDL210W	1.62	8.60E-09	*UGA4* – GABA–specific permease
YNR067C	1.60	8.74E-07	*ENG1* – Glucan endo–1,3–beta–D–glucosidase 1
YLR286C	1.58	1.85E-07	*CHIT* – Endochitinase
YNL160W	1.58	2.00E-13	*YGP1* – Protein Ygp1
YNL327W	1.54	2.36E-06	*EGT2* – Protein Egt2
YDR536W	1.50	4.22E-08	*STL1* – Sugar transporter Stl1
YGL008C	1.48	0.0032	*PMA1* – Plasma membrane ATPase 1

Table continued on next page.

Gene ID	Fold change	Adjusted p–value	Gene standard name and function
YNL142W	1.47	5.31E-05	*MEP2* – Ammonium transporter Mep2
YCR061W	1.46	0.0113	*YCV1* – Uncharacterized membrane protein
YML075C	1.46	0.0003	*HMDH1* – 3–hydroxy–3–methylglutaryl–coenzyme A reductase 1
YJL079C	1.45	0.0049	*PRY1* – Protein Pry1
YOL155C	1.44	0.0020	*HPF1* – Haze protective factor 1
YBR054W	1.44	5.71E-08	*YRO2* – Protein Yro2
YOL119C	1.44	8.84E-06	*MCH4* – Probable transporter Mch4
YMR034C	1.43	9.07E-06	*RCH1* – Solute carrier Rch1
YGR260W	1.43	5.16E-06	*TNA1* – High–affinity nicotinic acid transporter
YPL092W	1.42	9.57E-05	*SSU1* – Sulfite efflux pump Ssu1
YLR312C	1.42	0.0001	*ATG39* – Autophagy–related protein 39
YPL265W	1.42	1.70E-07	*DIP5* – Dicarboxylic amino acid permease
YNL066W	1.41	0.0064	*SUN4* – Probable secreted beta–glucosidase Sun4
YHL016C	1.40	0.0064	*DUR3* – Urea active transporter
YCR098C	1.40	0.0002	*GIT1* – Glycerophosphoinositol transporter 1
YGL055W	1.39	9.92E-06	*ACO1* – Acyl–CoA desaturase 1
YKL034W	1.37	0.0039	*TUL1* – Transmembrane E3 ubiquitin–protein ligase 1
YCR068W	1.37	6.12E-05	*ATG15* – Putative lipase Atg15

Table continued on next page.

Gene ID	Fold change	Adjusted p–value	Gene standard name and function
YOL101C	1.37	0.0046	*IZH4* – ADIPOR–like receptor Izh4
YDR205W	1.37	0.0035	*MSC2* – Probable zinc transporter Msc2
YKL187C	1.37	0.0051	*YKS7* – Uncharacterized protein
YOR348C	1.36	0.0008	*PUT4* – Proline-specific permease
YLL055W	1.36	0.0001	*YCT1* – High affinity cysteine transporter
YNL237W	1.36	0.0010	*YTP1* – Protein Ytp1
YKL198C	1.35	0.0056	*PTK1* – Serine/threonine-protein kinase Ptk1/Stk1
YJL042W	1.35	0.0243	*MHP1* – Map–homologous protein 1
YCR021C	1.35	0.0013	*HSP30* – 30 kDa heat shock protein
YCR017C	1.35	0.0003	*CWH43* – Protein Cwh43
YMR088C	1.34	0.0126	*VBA1* – Vacuolar basic amino acid transporter 1
YDL180W	1.34	0.0049	*YD180* – Uncharacterized membrane protein
YGR197C	1.33	0.0049	*SNG1* – Nitrosoguanidine resistance protein Sng1
YJL172W	1.33	0.0126	*CBPS* – Carboxypeptidase S
YMR266W	1.33	0.0162	*RSN1* – Uncharacterized protein Rsn1
novel.119	1.33	0.0049	*TPO3* – Polyamine transporter 3
YBR235W	1.32	0.0089	*VHC1* – Vacuolar cation–chloride cotransporter 1
YJR152W	1.32	0.0064	*DAL5* – Allantoate permease

Table continued on next page.

Gene ID	Fold change	Adjusted p–value	Gene standard name and function
YER052C	1.32	0.0030	*AK* – Aspartokinase
YOR049C	1.31	0.0323	*RSB1* – Sphingoid long–chain base transporter Rsb1
YGL186C	1.31	0.0032	*TPN1* – Vitamin B6 transporter Tpn1
YNL195C	1.31	0.0126	*YNT5* – Uncharacterized protein
YLR207W	1.31	0.0027	*HRD3* – ERAD–associated E3 ubiquitin–protein ligase component Hrd3
YLR299W	1.31	0.0044	*ECM38* – Glutathione hydrolase proenzyme
YLR001C	1.30	0.0366	*YL001* – Fas1 domain–containing protein
YBR229C	1.30	0.0191	*GLU2A* – Glucosidase 2 subunit alpha
YGL027C	1.30	0.0190	*CWH41* – Mannosyl–oligosaccharide glucosidase
YIL030C	1.30	0.0126	*DOA10* – ERAD–associated E3 ubiquitin–protein ligase Doa10
YDR033W	1.30	0.0051	*MRH1* – Protein Mrh1
YDR135C	1.30	0.0168	*YCFI* – Metal resistance protein Ycf1
YNR002C	1.30	0.0051	*ATO2* – Ammonia transport outward protein 2
YKL185W	1.29	0.0100	*ASH1* – Transcriptional regulatory protein Ash1
YIL090W	1.29	0.0101	*ICE2* – Protein Ice2
YLR152C	1.29	0.0101	*YL152* – Uncharacterized transporter

Table continued on next page.

Gene ID	Fold change	Adjusted p–value	Gene standard name and function
YGL146C	1.28	0.0352	*RRT6* – Regulator of rDNA transcription protein 6
YPR026W	1.28	0.0243	*ATH1* – Periplasmic acid trehalase Atc1
YDL237W	1.28	0.0100	*AIM6* – Altered inheritance of mitochondria protein 6
YGL117W	1.28	0.0318	*YGL7* – Uncharacterized protein
YOR002W	1.28	0.0100	*ALG6* – Dolichyl pyrophosphate Man9GlcNAc2 alpha–1,3–glucosyltransferase
YKL217W	1.28	0.0027	*JEN1* – Carboxylic acid transporter protein homolog
YJL045W	1.28	0.0243	*SDHX* – Succinate dehydrogenase [ubiquinone] flavoprotein subunit 2, mitochondrial
YDR284C	1.28	0.0082	*DPP1* – Diacylglycerol pyrophosphate phosphatase 1
YOL058W	1.28	0.0134	*ASSY* – Argininosuccinate synthase
YJR106W	1.28	0.0467	*ECM27* – Protein Ecm27
YDR090C	1.27	0.0076	*YD090* – Uncharacterized membrane protein
YJL062W	1.27	0.0182	*GPI7* – GPI ethanolamine phosphate transferase 2
YGL006W	1.27	0.0272	*ATC2* – Calcium–transporting ATPase 2
YMR221C	1.27	0.0185	*FMP42* – Protein Fmp42
YCR010C	1.27	0.0058	*ADY2* – Accumulation of dyads protein 2

Table continued on next page.

Gene ID	Fold change	Adjusted p–value	Gene standard name and function
YNL194C	1.27	0.0114	*YNT4* – Uncharacterized plasma membrane protein
YDL093W	1.26	0.0101	*PMT5* – Dolichyl–phosphate-mannose-–protein mannosyltransferase 5
YMR243C	1.26	0.0070	*ZRC1* – Vacuolar zinc transporter Zrc1
YDR492W	1.26	0.0058	*IZH1* – ADIPOR–like receptor Izh1
YDR307W	1.26	0.0185	*PMT7* – Probable dolichyl–phosphate-mannose-–protein mannosyltransferase 7
YPL274W	1.26	0.0318	*SAM3* – S–adenosylmethionine permease Sam3
YOR303W	1.26	0.0027	*CARA* – Carbamoyl phosphate synthase arginine-specific small chain
YDL054C	1.26	0.0166	*MCH1* – Probable transporter Mch1
YBR241C	1.26	0.0063	*YB91* – Probable metabolite transport protein
YJL163C	1.26	0.0162	*YJQ3* – Uncharacterized membrane protein
YGR121C	1.26	0.0244	*MEP1* – Ammonium transporter Mep1
YDR483W	1.26	0.0092	*KRE2* – Glycolipid 2–alpha–mannosyltransferase
YBR072W	1.26	0.0207	*HSP26* – Heat shock protein 26
YPL026C	1.26	0.0194	*SKS1* – Serine/threoninE-protein kinase Sks1
YOL013C	1.25	0.0116	*HRD1* – ERAD–associated E3 ubiquitin–protein ligase Hrd1

Table continued on next page.

Gene ID	Fold change	Adjusted p–value	Gene standard name and function
YMR297W	1.25	0.0100	*CBPY* – Carboxypeptidase Y
YJR013W	1.25	0.0443	*GPI14* – GPI mannosyltransferase 1
YGR060W	1.25	0.0058	*ERG25* – C–4 methylsterol oxidase Erg25
YHR039C	1.25	0.0144	*MSC7* – Putative aldehyde dehydrogenase-like protein
YBR069C	1.25	0.0130	*TAT1* – Valine/tyrosine/tryptophan amino–acid permease 1
YKL004W	1.25	0.0318	*AUR1* – Inositol phosphorylceramide synthase catalytic subunit Aur1
YKL140W	1.25	0.0131	*TGL1* – Sterol esterase Tgl1
YNL268W	1.24	0.0126	*LYP1* – Lysine-specific permease
YML128C	1.24	0.0090	*MSC1* – Meiotic sister chromatid recombination protein 1
YER056C	1.24	0.0243	*FCY2* – Purine-cytosine permease Fcy2
YPR083W	1.24	0.0156	*MDM36* – Mitochondrial distribution and morphology protein 36
YHR202W	1.24	0.0131	*YH02* – Uncharacterized protein
YKR067W	1.24	0.0162	*GPT2* – Glycerol–3–phosphate O–acyltransferase 2
YBR043C	1.24	0.0489	*QDR3* – Quinidine resistance protein 3
YCL025C	1.24	0.0165	*AGP1* – General amino acid permease Agp1
YAL062W	1.24	0.0194	*DHE5* – NADP–specific glutamate dehydrogenase 2

Table continued on next page.

Gene ID	Fold change	Adjusted p–value	Gene standard name and function
YGL022W	1.24	0.0372	*STT3* – Dolichyl–diphosphooligosaccharide–protein glycosyltransferase subunit Stt3
YKL207W	1.24	0.0296	*EMC3* – ER membrane protein complex subunit 3
novel.460	1.24	0.0230	*G3P1* – Glyceraldehyde-3–phosphate dehydrogenase 1
YLR056W	1.23	0.0162	*ERG3* – Delta(7)–sterol 5(6)–desaturase Erg3
YDR233C	1.23	0.0100	*RTN1* – Reticulon–like protein 1
YOR321W	1.23	0.0415	*PMT3* – Dolichyl–phosphate-mannose-–protein mannosyltransferase 3
YNL280C	1.23	0.0266	*ERG24* – Delta(14)–sterol reductase Erg24
YDR481C	1.23	0.0461	*PPB* – Repressible alkaline phosphatase
YMR015C	1.23	0.0084	*ERG5* – C–22 sterol desaturase Erg5
YDR456W	1.23	0.0372	*NHX1* – Endosomal/prevacuolar sodium/hydrogen exchanger
YBR110W	1.23	0.0279	*ALG1* – Chitobiosyldiphosphodolichol beta–mannosyltransferase
YFL041W	1.23	0.0243	*FET5* – Iron transport multicopper oxidase Fet5
YBR067C	1.22	0.0105	*TIP1* – Temperature shock–inducible protein 1
YHR188C	1.22	0.0243	*GPI16* – GPI transamidase component Gpi16
YIL088C	1.22	0.0296	*AVT7* – Vacuolar amino acid transporter 7

Table continued on next page.

Gene ID	Fold change	Adjusted p–value	Gene standard name and function
YDL204W	1.22	0.0126	*RTN2* – Reticulon–like protein 2
YGR284C	1.22	0.0372	*ERV29* – ER–derived vesicles protein Erv29
YGL077C	1.22	0.0256	*HNM1* – Choline transporter
YOR316C	1.22	0.0201	*COT1* – Vacuolar zinc transporter Cot1
YNL130C	1.21	0.0427	*CPT1* – Cholinephosphotransferase 1
novel.318	1.21	0.0286	*ATR* – Serine/threoninE-protein kinase Mec1
YBR015C	1.21	0.0489	*MNN2* – Alpha–1,2–mannosyltransferase Mnn2
YBR036C	1.21	0.0369	*CSG2* – Mannosyl phosphorylinositol ceramide synthase regulatory protein Csg2
YHR132C	1.21	0.0244	*ECM14* – Inactive metallocarboxypeptidase Ecm14
YNL115C	1.21	0.0467	*YNL5* – Uncharacterized vacuolar membrane protein
YBR286W	1.19	0.0374	*APE3* – Aminopeptidase Y
YGL253W	0.84	0.0468	*HXKB* – Hexokinase-2
YKL195W	0.84	0.0443	*MIA40* – Mitochondrial intermembrane space import and assembly protein 40
YFL016C	0.82	0.0126	*MDJ1* – DnaJ homolog 1, mitochondrial
YHR137W	0.82	0.0472	*ARO9* – Aromatic amino acid aminotransferase 2
YOR187W	0.81	0.0073	*EFTU* – Elongation factor Tu, mitochondrial

Table continued on next page.

Gene ID	Fold change	Adjusted p–value	Gene standard name and function
YPR047W	0.81	0.0326	*SYFM* – Phenylalanine–tRNA ligase, mitochondrial
YIL117C	0.81	0.0492	*PRM5* – Pheromone-regulated membrane protein 5
YGR161C	0.81	0.0243	*RTS3* – Protein phosphatase type 2A regulatory subunit Rts3
YDR381W	0.81	0.0410	*YRA1* – RNA annealing protein Yra1
YER077C	0.81	0.0358	*MRX1* – MIOREX complex component 1
YOL096C	0.79	0.0382	*COQ3* – Ubiquinone biosynthesis O–methyltransferase, mitochondrial
YMR193W	0.79	0.0131	*RM24* – Large ribosomal subunit protein bL28m
YLR151C	0.79	0.0272	*PCD1* – Peroxisomal coenzyme A diphosphatase 1, peroxisomal
YPR158W	0.79	0.0366	*CUR1* – Curing of [URE3] protein 1
YMR024W	0.79	0.0101	*RM03* – Large ribosomal subunit protein mL44
YJR094W_A	0.78	0.0272	*RL43A* – Large ribosomal subunit protein eL43A
YLR069C	0.78	0.0027	*EFGM* – Elongation factor G, mitochondrial
YKL216W	0.77	0.0020	*PYRD* – Dihydroorotate dehydrogenase (fumarate)
YHR209W	0.77	0.0126	*CRG1* – Probable S–adenosylmethioninE-dependent methyltransferase Crg1
YPR101W	0.77	0.0045	*SN309* – Pre-mRNA–splicing factor Snt309

Table continued on next page.

Gene ID	Fold change	Adjusted p–value	Gene standard name and function
YBL078C	0.75	0.0057	*ATG8* – Autophagy–related protein 8
YOR195W	0.75	0.0011	*SLK19* – Kinetochore protein Slk19
novel.205	0.73	0.0316	*YB230* – Uncharacterized protein
YOR131C	0.73	0.0058	*YOR31* – Uncharacterized hydrolase
YLR160C	0.62	4.63E-09	*ASP21* – L–asparaginase 2–1
