# Supplementary material for: Inherited long telomeres induce a genome-wide transcriptional response in budding yeast
Source: Genetics. 2026 Jul 3;234(2):iyag174. doi: 10.1093/genetics/iyag174 (PMC13643770; doi:10.1093/genetics/iyag174)

File S2: Analyses including the outlier RNA-seq replicate c56z1 (*WT/rif2Δ*)

I. Lists of differentially expressed genes (DEGs) across the three pairwise comparisons

II. Heatmap of significantly differentially expressed genes (adjusted $p \leq 0.05$) across the three pairwise comparisons

III. Venn diagram illustrating the overlap between significantly differentially expressed genes (adjusted $p \leq 0.05$) across the three pairwise comparisons

IV. Gene ontology analysis of the 59 DEGs shared across the three pairwise comparisons

V. Genomic position and Rap1-binding status of the 59 DEGs

VI. Relationship between $|\log_2 FC|$ and distance from the nearest telomere (kb) for the 59 DEGs

VII. Per-gene changes in $|\log_2 FC|$ across long-telomere strains for the 59 DEGs

I. Lists of differentially expressed genes across the three pairwise comparisons

1) *WT/WT_long1* vs *WT/WT*

Gene ID	Fold change	Adjusted p-value	Gene standard name and function
YDL223C	1.89	2.21E-12	*HBT1* – Protein Hbt1
YHR139C	1.73	0.0167	*SS100* – Sporulation-specific wall maturation protein
YDL222C	1.61	3.42E-20	*FMP45* – SUR7 family protein Fmp45
YJL116C	1.58	0.0001	*NCA3* – Beta-glucosidase-like protein Nca3, mitochondrial
YBR296C	1.55	0.0032	*PHO89* – Phosphate permease Pho89
YDL210W	1.54	9.21E-06	*UGA4* – GABA-specific permease
YHR096C	1.46	3.07E-07	*HXT5* – Probable glucose transporter Hxt5
YNL160W	1.46	4.44E-13	*YGP1* – Protein Ygp1
YCR061W	1.45	0.0032	*YCV1* – Uncharacterized membrane protein
YGR249W	1.45	0.0151	*MGA1* – Protein Mga1
YPL036W	1.44	0.0034	*PMA2* – Plasma membrane ATPase 2
YPL223C	1.43	3.67E-05	*GRE1* – Protein Gre1
YNL195C	1.42	9.85E-05	*YNT5* – Uncharacterized protein Ynl195c
YDL214C	1.41	0.0001	*PRR2* – Serine/threonine-protein kinase Prr2
YOL084W	1.40	0.0003	*PHM7* – Phosphate metabolism protein 7
YHL016C	1.39	0.0003	*DUR3* – Urea active transporter

Gene ID	Fold change	Adjusted p-value	Gene standard name and function
YBR117C	1.38	0.0014	*TKT2* – Transketolase 2
YBL075C	1.36	0.0014	*HSP73* – Heat shock protein Ssa3
YBR054W	1.36	9.09E-08	*YRO2* – Protein Yro2
YML123C	1.36	0.0007	*PHO84* – Inorganic phosphate transporter Pho84
YMR306W	1.35	0.0032	*FKS3* – 1,3-beta-glucan synthase component Fks3
YGR052W	1.35	0.0341	*FMP48* – Probable serine/threonine-protein kinase Fmp48
YML116W	1.34	0.0048	*ATR1* – Aminotriazole resistance protein
YML128C	1.34	6.09E-07	*MSC1* – Meiotic sister chromatid recombination protein 1
YNL142W	1.33	0.0002	*MEP2* – Ammonium transporter Mep2
YOR328W	1.33	0.0001	*PDR10* – ATP-dependent permease Pdr10
YPR026W	1.33	0.0019	*ATH1* – Periplasmic acid trehalase Atc1
YDR406W	1.33	0.0271	*PDR15* – ATP-dependent permease Pdr15
YLR413W	1.33	0.0038	*YL413* – Cell membrane protein
YNL194C	1.33	1.21E-06	*YNT4* – Uncharacterized plasma membrane protein
YDR205W	1.32	0.0003	*MSC2* – Probable zinc transporter Msc2
novel.28	1.32	0.0035	*YB21B* – Transposon Ty2-B Gag-Pol polyprotein
YLR450W	1.32	0.0151	*HMDH2* – 3-hydroxy-3-methylglutaryl-coenzyme A reductase 2

Gene ID	Fold change	Adjusted p-value	Gene standard name and function
YJR094C	1.32	0.0012	*IME1* – Meiosis-inducing protein 1
YNL014W	1.32	0.0175	*EF3B* – Elongation factor 3b
YOL119C	1.31	6.05E-05	*MCH4* – Probable transporter Mch4
YGL055W	1.31	7.74E-05	*ACO1* – Acyl-CoA desaturase 1
YGL008C	1.31	0.0394	*PMA1* – Plasma membrane ATPase 1
YKL187C	1.30	0.0034	*YKS7* – Uncharacterized protein
YMR266W	1.30	0.0056	*RSN1* – Uncharacterized protein Rsn1
YBR235W	1.30	0.0029	*VHC1* – Vacuolar cation-chloride cotransporter 1
YJR106W	1.30	0.0046	*ECM27* – Protein Ecm27
novel.9	1.29	0.0213	*YP202* – Putative uncharacterized protein
YJL045W	1.29	0.0035	*SDHX* – Succinate dehydrogenase [ubiquinone] flavoprotein subunit 2, mitochondrial
YJL172W	1.29	0.0116	*CBPS* – Carboxypeptidase S
novel.17	1.29	0.0254	*YL066* – Y' element ATP-dependent helicase
YDR420W	1.28	0.0303	*HKR1* – Signaling mucin Hkr1
YPL006W	1.28	0.0420	*NPC1* – NPC intracellular sterol transporter 1-related protein 1
YIL030C	1.28	0.0032	*DOA10* – ERAD-associated E3 ubiquitin-protein ligase Doa10
YER150W	1.28	0.0294	*SPI1* – Uncharacterized cell wall protein Spi1
YCR021C	1.28	0.0048	*HSP30* – 30 kDa heat shock protein

Gene ID	Fold change	Adjusted p-value	Gene standard name and function
YGR143W	1.28	0.0146	*SKN1* – Beta-glucan synthesis-associated protein Skn1
YPL275W	1.28	0.0197	*FDH2* – Truncated formate dehydrogenase 2
YGR281W	1.28	0.0428	*YOR1* – Oligomycin resistance ATP-dependent permease Yor1
YCR032W	1.27	0.0175	*BPH1* – Beige protein homolog 1
YDL204W	1.27	3.41E-05	*RTN2* – Reticulon-like protein 2
novel.134	1.27	0.0029	*TPO3* – Polyamine transporter 3
YCR068W	1.27	0.0002	*ATG15* – Putative lipase Atg15
YKL034W	1.27	0.0173	*TUL1* – Transmembrane E3 ubiquitin-protein ligase 1
YKL050C	1.26	0.0471	*YKF0* – Uncharacterized protein
YML075C	1.26	0.0243	*HMDH1* – 3-hydroxy-3-methylglutaryl-coenzyme A reductase 1
YMR034C	1.26	0.0005	*RCH1* – Solute carrier Rch1
YIL119C	1.26	0.0195	*RPI1* – Negative RAS protein regulator protein
YLL015W	1.26	0.0489	*BPT1* – Bile pigment transporter 1
YER096W	1.26	0.0300	*SHC1* – Protein Shc1
YPL137C	1.26	0.0390	*GIP3* – GLC7-interacting protein 3
novel.91	1.26	0.0418	*TPO3* – Polyamine transporter 3
YBR072W	1.26	0.0250	*HSP26* – Heat shock protein 26
YDL199C	1.25	0.0418	*YD199* – Putative metabolite transport protein
YLL055W	1.25	0.0087	*YCT1* – High affinity cysteine transporter

Gene ID	Fold change	Adjusted p-value	Gene standard name and function
YPL265W	1.25	8.56E-05	*DIP5* – Dicarboxylic amino acid permease
YDR135C	1.25	0.0270	*YCFI* – Metal resistance protein Ycf1
YHR202W	1.25	0.0006	*YH02* – Uncharacterized protein
YHR056C	1.24	0.0175	*RSC30* – Chromatin structure-remodeling complex protein Rsc30
YOL101C	1.24	0.0157	*IZH4* – ADIPOR-like receptor Izh4
YLR152C	1.24	0.0080	*YL152* – Uncharacterized transporter
YGR260W	1.24	0.0034	*TNA1* – High-affinity nicotinic acid transporter
YCR017C	1.24	0.0034	*CWH43* – Protein Cwh43
YGL027C	1.24	0.0390	*CWH41* – Mannosyl-oligosaccharide glucosidase
YJL005W	1.23	0.0465	*CYAA* – Adenylate cyclase
YLR312C	1.23	0.0051	*ATG39* – Autophagy-related protein 39
YBL004W	1.23	0.0214	*UTP20* – U3 small nucleolar RNA-associated protein 20
YOR391C	1.23	0.0214	*HSP33* – Probable glutathione-independent glyoxalase Hsp33
YNL237W	1.23	0.0081	*YTP1* – Protein Ytp1
YLR371W	1.23	0.0216	*ROM2* – RHO1 GDP-GTP exchange protein 2
YHR080C	1.23	0.0385	*LAM4* – Membrane-anchored lipid-binding protein Lam4
YBR241C	1.23	0.0007	*YB91* – Probable metabolite transport protein

Gene ID	Fold change	Adjusted p-value	Gene standard name and function
YGL006W	1.22	0.0276	*ATC2* – Calcium-transporting ATPase 2
novel.7	1.22	0.0380	*YMN3* – Y' element ATP-dependent helicase
YLR207W	1.22	0.0048	*HRD3* – ERAD-associated E3 ubiquitin-protein ligase component Hrd3
novel.13	1.21	0.0163	*TY1AB* – Transposon TyH3 Gag-Pol polyprotein
YGR121C	1.21	0.0175	*MEP1* – Ammonium transporter Mep1
YJL163C	1.21	0.0157	*YJQ3* – Uncharacterized membrane protein
YDR422C	1.21	0.0481	*SIP1* – SNF1 protein kinase subunit beta-1
YIL162W	1.21	0.0175	*INV2* – Invertase 2
YDL237W	1.21	0.0197	*AIM6* – Altered inheritance of mitochondria protein 6
novel.22	1.21	0.0366	*YD22B* – Transposon Ty2-DR2 Gag-Pol polyprotein
YKL217W	1.21	0.0038	*JEN1* – Carboxylic acid transporter protein homolog
YLR299W	1.21	0.0173	*ECM38* – Glutathione hydrolase proenzyme
YDR307W	1.20	0.0215	*PMT7* – Probable dolichyl-phosphate-mannose--protein mannosyltransferase 7
YGL186C	1.20	0.0175	*TPN1* – Vitamin B6 transporter Tpn1
YBR067C	1.20	0.0014	*TIP1* – Temperature shock-inducible protein 1
YKL140W	1.20	0.0300	*TGL1* – Sterol esterase Tgl1
YDL054C	1.20	0.0163	*MCH1* – Probable transporter Mch1

Gene ID	Fold change	Adjusted p-value	Gene standard name and function
YHR039C	1.20	0.0412	*MSC7* – Putative aldehyde dehydrogenase-like protein
YNL268W	1.19	0.0166	*LYP1* – Lysine-specific permease
YNL115C	1.19	0.0186	*YNL5* – Uncharacterized vacuolar membrane protein
YKL008C	1.19	0.0247	*LAC1* – Sphingosine N-acyltransferase Lac1
YBR286W	1.18	0.0095	*APE3* – Aminopeptidase Y
YOL013C	1.18	0.0197	*HRD1* – ERAD-associated E3 ubiquitin-protein ligase Hrd1
YOR002W	1.18	0.0443	*ALG6* – Dolichyl pyrophosphate Man9GlcNAc2 alpha-1,3-glucosyltransferase
YGR060W	1.18	0.0164	*ERG25* – C-4 methylsterol oxidase Erg25
YDL128W	1.17	0.0465	*VCX1* – Vacuolar calcium ion transporter
YDR492W	1.17	0.0224	*IZH1* – ADIPOR-like receptor Izh1
novel.358	1.17	0.0270	*ATR* – Serine/threonine-protein kinase Mec1
YDR536W	1.17	0.0443	*STL1* – Sugar transporter Stl1
YDR483W	1.17	0.0418	*KRE2* – Glycolipid 2-alpha-mannosyltransferase
novel.23	1.17	0.0415	*TY1AB* – Transposon TyH3 Gag-Pol polyprotein
YHR132C	1.15	0.0421	*ECM14* – Inactive metallocarboxypeptidase Ecm14
YDR116C	0.86	0.0494	*RM01* – Large ribosomal subunit protein uL1m
YFL045C	0.85	0.0394	*PMM* – Phosphomannomutase

Gene ID	Fold change	Adjusted p-value	Gene standard name and function
YER077C	0.85	0.0494	*MRX1* – MIOREX complex component 1
YJR076C	0.85	0.0493	*CDC11* – Cell division control protein 11
YJR133W	0.85	0.0370	*XPT1* – Xanthine phosphoribosyltransferase 1
YKL085W	0.85	0.0280	*MDHM* – Malate dehydrogenase, mitochondrial
YGR165W	0.85	0.0390	*RT35* – Small ribosomal subunit protein mS45
YML124C	0.85	0.0261	*TBA3* – Tubulin alpha-3 chain
YOR327C	0.84	0.0494	*SNC2* – Synaptobrevin homolog 2
YNL067W	0.84	0.0371	*RL9B* – Large ribosomal subunit protein uL6B
YDR316W	0.84	0.0197	*OMS1* – Methyltransferase Oms1, mitochondrial
YNL284C	0.84	0.0452	*RM10* – Large ribosomal subunit protein uL15m
YBR010W	0.84	0.0480	*H3* – Histone H3
YFL016C	0.84	0.0045	*MDJ1* – DnaJ homolog 1, mitochondrial
YMR024W	0.84	0.0226	*RM03* – Large ribosomal subunit protein mL44
YKR006C	0.84	0.0215	*RM13* – Large ribosomal subunit protein mL50
YPL097W	0.83	0.0321	*SYYM* – Tyrosine--tRNA ligase, mitochondrial
YGR082W	0.83	0.0201	*TOM20* – Mitochondrial import receptor subunit Tom20
YJR101W	0.83	0.0245	*RT26* – Small ribosomal subunit protein mS42
YPL098C	0.83	0.0412	*MGR2* – Protein Mgr2

Gene ID	Fold change	Adjusted p-value	Gene standard name and function
YGL068W	0.83	0.0473	*MNP1* – Large ribosomal subunit protein bL12m
YOL053W	0.83	0.0189	*AIM39* – Altered inheritance of mitochondria protein 39, mitochondrial
YNL073W	0.83	0.0236	*SYKM* – Lysine--tRNA ligase, mitochondrial
YLR069C	0.83	0.0168	*EFGM* – Elongation factor G, mitochondrial
YDR337W	0.82	0.0182	*RT28* – Small ribosomal subunit protein uS15m
YDR487C	0.82	0.0189	*RIB3* – 3,4-dihydroxy-2-butanone 4-phosphate synthase
YPL256C	0.82	0.0473	*CG12* – G1/S-specific cyclin Cln2
YKL195W	0.82	0.0008	*MIA40* – Mitochondrial intermembrane space import and assembly protein 40
YKR013W	0.82	0.0314	*PRY2* – Protein Pry2
YJL158C	0.82	0.0126	*CIS3* – Cell wall mannoprotein Cis3
YLR168C	0.82	0.0076	*UPS2* – Protein Ups2, mitochondrial
YKL216W	0.82	0.0024	*PYRD* – Dihydroorotate dehydrogenase (fumarate)
YLR204W	0.82	0.0138	*COX24* – Small ribosomal subunit protein mS38
YDL235C	0.82	0.0465	*YPD1* – Phosphorelay intermediate protein Ypd1
YIL070C	0.81	0.0087	*MAM33* – Mitochondrial acidic protein Mam33
YBL038W	0.81	0.0191	*RM16* – Large ribosomal subunit protein uL16m
YMR193W	0.81	0.0056	*RM24* – Large ribosomal subunit protein bL28m

Gene ID	Fold change	Adjusted p-value	Gene standard name and function
YNR022C	0.81	0.0278	*RM50* – Large ribosomal subunit protein bL9m
YOR187W	0.80	5.34E-05	*EFTU* – Elongation factor Tu, mitochondrial
YPR158W	0.80	0.0064	*CUR1* – Curing of [URE3] protein 1
YBL090W	0.80	0.0424	*RT21* – Small ribosomal subunit protein bS21m
YNL289W	0.80	0.0017	*PCL1* – PHO85 cyclin-1
YJR048W	0.80	0.0032	*CYC1* – Cytochrome c isoform 1
YNL300W	0.79	0.0167	*TOS6* – Protein Tos6
YMR157C	0.79	0.0157	*AIM36* – Altered inheritance of mitochondria protein 36
YFR001W	0.79	0.0189	*LOC1* – 60S ribosomal subunit assembly/export protein Loc1
YBR009C	0.78	0.0011	*H4* – Histone H4
YBL003C	0.78	0.0054	*H2A2* – Histone H2A.2
YNR049C	0.76	0.0163	*MSO1* – Protein Mso1
YHR059W	0.75	0.0020	*FYV4* – Small ribosomal subunit protein mS41
novel.376	0.74	0.0226	*COX17* – Cytochrome c oxidase copper chaperone
YNL030W	0.74	3.78E-05	*H4* – Histone H4
YDR478W	0.71	0.0416	*RMRP* – Ribonuclease MRP protein subunit Snm1
YBL002W	0.67	3.67E-05	*H2B2* – Histone H2B.2
YPL144W	0.66	0.0241	*POC4* – Proteasome chaperone 4

Table continued on next page.

2) *WT/WT_long2* vs *WT/WT*

Gene ID	Fold change	Adjusted p-value	Gene standard name and function
YOR338W	2.13	0.0346	*YO338* – SWIRM domain-containing protein Yor338w
YDL223C	1.91	0.0226	*HBT1* – Protein Hbt1
novel.38	1.83	0.0109	*SSF1* – Ribosome biogenesis protein Ssf1
YPL036W	1.76	0.0029	*PMA2* – Plasma membrane ATPase 2
novel.361	1.74	0.0005	*DBP2* – ATP-dependent RNA helicase Dbp2
YBR105C	1.70	0.0012	*GID4* – Vacuolar import and degradation protein 24
YNL160W	1.58	1.93E-08	*YGP1* – Protein Ygp1
YER187W	1.58	0.0207	*YE17* – Uncharacterized protein Yer187w
YBR054W	1.57	0.0005	*YRO2* – Protein Yro2
YBR296C	1.55	0.0021	*PHO89* – Phosphate permease Pho89
YGR079W	1.55	0.0028	*YG2G* – Putative uncharacterized protein Ygr079w
YHR096C	1.54	0.0005	*HXT5* – Probable glucose transporter Hxt5
YOR107W	1.54	0.0067	*RGS2* – Regulator of G-protein signaling 2
YOR359W	1.53	0.0194	*VTS1* – RNA-binding protein Vts1
novel.501	1.52	0.0008	-
YKR075C	1.51	0.0011	*YK55* – Uncharacterized protein Ykr075c
YBR238C	1.50	0.0053	*RMD9L* – RNA-binding protein RMD9-like, mitochondrial

**Gene ID**	**Fold change**	**Adjusted p-value**	**Gene standard name and function**
YER127W	1.49	0.0267	*LCP5* – U3 small nucleolar ribonucleoprotein protein Lcp5
YGL008C	1.46	0.0031	*PMA1* – Plasma membrane ATPase 1
YML116W	1.45	0.0024	*ATR1* – Aminotriazole resistance protein
YMR095C	1.44	0.0058	*SNO1* – Pyridoxal 5'-phosphate synthase subunit Sno1
YDL048C	1.44	0.0008	*STP4* – Zinc finger protein Stp4
YLR413W	1.43	0.0058	*YL413* – Cell membrane protein Ylr413w
YMR306W	1.43	0.0012	*FKS3* – 1,3-beta-glucan synthase component Fks3
YDL210W	1.42	0.0005	*UGA4* – GABA-specific permease
novel.134	1.41	0.0005	*TPO3* – Polyamine transporter 3
YOR360C	1.41	0.0051	*PDE2* – 3',5'-cyclic-nucleotide phosphodiesterase 2
YGL186C	1.41	0.0005	*TPN1* – Vitamin B6 transporter Tpn1
YGL028C	1.40	0.0029	*SCW11* – Probable family 17 glucosidase Scw11
YIL053W	1.40	0.0216	*GPP1* – Glycerol-1-phosphate phosphohydrolase 1
YBR104W	1.40	0.0401	*YMC2* – Carrier protein YMC2, mitochondrial
YNL234W	1.40	0.0285	*YNX4* – Uncharacterized globin-like protein Ynl234w
YOL084W	1.40	0.0072	*PHM7* – Phosphate metabolism protein 7
YPL026C	1.40	0.0058	*SKS1* – Serine/threonine-protein kinase Sks1

Gene ID	Fold change	Adjusted p-value	Gene standard name and function
YER150W	1.39	0.0199	*SPI1* – Uncharacterized cell wall protein Spi1
YCR061W	1.39	0.0263	*YCV1* – Uncharacterized membrane protein Ycr061w
YDR277C	1.39	0.0029	*MTH1* – Protein Mth1
YML123C	1.38	0.0005	*PHO84* – Inorganic phosphate transporter Pho84
YDR465C	1.38	0.0278	*RMT2* – Protein arginine N-methyltransferase 2
YJL116C	1.37	0.0332	*NCA3* – Beta-glucosidase-like protein NCA3, mitochondrial
YOL119C	1.37	0.0008	*MCH4* – Probable transporter Mch4
YIL162W	1.37	0.0161	*INV2* – Invertase 2
YDR406W	1.37	0.0218	*PDR15* – ATP-dependent permease Pdr15
YGL055W	1.36	0.0011	*ACO1* – Acyl-CoA desaturase 1
YBL075C	1.36	0.0282	*HSP73* – Heat shock protein Ssa3
YHR170W	1.34	0.0248	*NMD3* – 60S ribosomal export protein Nmd3
novel.91	1.34	0.0199	*TPO3* – Polyamine transporter 3
YCR021C	1.34	0.0285	*HSP30* – 30 kDa heat shock protein
YDL063C	1.34	0.0158	*SYO1* – Synchronized import protein 1
YNL014W	1.34	0.0121	*EF3B* – Elongation factor 3b
YNL195C	1.33	0.0109	*YNT5* – Uncharacterized protein Ynl195c
YDR527W	1.33	0.0270	*RBA50* – RNA polymerase II-associated protein Rba50

**Gene ID**	**Fold change**	**Adjusted p-value**	**Gene standard name and function**
YPL043W	1.33	0.0242	*NOP4* – Nucleolar protein 4
YKL187C	1.32	0.0218	*YKS7* – Uncharacterized protein Ykl187c
YGR052W	1.32	0.0162	*FMP48* – Probable serine/threonine-protein kinase Fmp48
YDR075W	1.32	0.0281	*PP4C* – Serine/threonine-protein phosphatase 4 catalytic subunit
YMR085W	1.32	0.0440	*YM084* – Putative glutamine--fructose-6-phosphate aminotransferase [isomerizing]
YDL204W	1.32	0.0203	*RTN2* – Reticulon-like protein 2
YML128C	1.32	0.0061	*MSC1* – Meiotic sister chromatid recombination protein 1
YNR067C	1.32	0.0024	*ENG1* – Glucan endo-1,3-beta-D-glucosidase 1
YDL214C	1.32	0.0086	*PRR2* – Serine/threonine-protein kinase Prr2
YKL034W	1.31	0.0109	*TUL1* – Transmembrane E3 ubiquitin-protein ligase 1
YGR191W	1.31	0.0026	*HIP1* – Histidine permease
YIL030C	1.31	0.0067	*DOA10* – ERAD-associated E3 ubiquitin-protein ligase Doa10
YJL033W	1.31	0.0135	*DBP4* – ATP-dependent RNA helicase Dbp4
YKR098C	1.31	0.0039	*UBP11* – Ubiquitin carboxyl-terminal hydrolase 11
YNL042W	1.31	0.0463	*BOP3* – Protein Bop3
YNL124W	1.31	0.0305	*NAF1* – H/ACA ribonucleoprotein complex non-core subunit Naf1
YOR049C	1.31	0.0384	*RSB1* – Sphingoid long-chain base transporter Rsb1

Gene ID	Fold change	Adjusted p-value	Gene standard name and function
YML118W	1.31	0.0189	*NGL3* – Probable RNA exonuclease Ngl3
YOR337W	1.30	0.0095	*TEA1* – TY1 enhancer activator
YDR205W	1.30	0.0091	*MSC2* – Probable zinc transporter Msc2
YDR247W	1.30	0.0109	*VHS1* – Serine/threonine-protein kinase Vhs1
YLR450W	1.30	0.0395	*HMDH2* – 3-hydroxy-3-methylglutaryl-coenzyme A reductase 2
YCR068W	1.30	0.0053	*ATG15* – Putative lipase Atg15
YHR052W	1.30	0.0297	*CIC1* – Proteasome-interacting protein Cic1
YHL016C	1.30	0.0199	*DUR3* – Urea active transporter
YPL126W	1.30	0.0122	*UTP17* – NET1-associated nuclear protein 1
YER052C	1.30	0.0017	*AK* – Aspartokinase
YDR101C	1.30	0.0490	*ARX1* – Probable metalloprotease Arx1
YGR143W	1.30	0.0161	*SKN1* – Beta-glucan synthesis-associated protein Skn1
YJL172W	1.30	0.0263	*CBPS* – Carboxypeptidase S
YHR143W	1.29	0.0278	*DSE2* – Protein Dse2
YMR096W	1.29	0.0449	*SNZ1* – Pyridoxal 5'-phosphate synthase subunit Snz1
YMR189W	1.29	0.0297	*GCSP* – Glycine dehydrogenase (decarboxylating), mitochondrial
YDR033W	1.29	0.0199	*MRH1* – Protein Mrh1
YBR235W	1.29	0.0122	*VHC1* – Vacuolar cation-chloride cotransporter 1

Gene ID	Fold change	Adjusted p-value	Gene standard name and function
YMR266W	1.28	0.0285	*RSN1* – Uncharacterized protein Rsn1
YLL010C	1.28	0.0278	*PSR1* – Phosphatase Psr1
novel.28	1.28	0.0399	*YB21B* – Transposon Ty2-B Gag-Pol polyprotein
YPL092W	1.28	0.0458	*SSU1* – Sulfite efflux pump Ssu1
YDR496C	1.28	0.0202	*PUF6* – Pumilio homology domain family member 6
YOR204W	1.28	0.0278	*DED1* – ATP-dependent RNA helicase Ded1
YPL137C	1.28	0.0105	*GIP3* – GLC7-interacting protein 3
YOL158C	1.28	0.0425	*ENB1* – Siderophore iron transporter Enb1
YGR197C	1.27	0.0157	*SNG1* – Nitrosoguanidine resistance protein Sng1
YHR150W	1.27	0.0203	*PEX28* – Peroxisomal membrane protein Pex28
YNR075W	1.27	0.0401	*COS10* – Protein Cos10
YMR319C	1.27	0.0039	*FET4* – Low-affinity Fe(2+) transport protein
YBR114W	1.27	0.0126	*RAD16* – DNA repair protein Rad16
YDR135C	1.27	0.0251	*YCFI* – Metal resistance protein Ycf1
YMR034C	1.27	0.0029	*RCH1* – Solute carrier Rch1
YER056C	1.27	0.0058	*FCY2* – Purine-cytosine permease Fcy2
YOR391C	1.27	0.0203	*HSP33* – Probable glutathione-independent glyoxalase Hsp33
YHR202W	1.27	0.0067	*YH02* – Uncharacterized protein Yhr202w

Gene ID	Fold change	Adjusted p-value	Gene standard name and function
YBR067C	1.27	0.0024	*TIP1* – Temperature shock-inducible protein 1
YIL130W	1.26	0.0058	*ASG1* – Activator of stress genes 1
YHR029C	1.26	0.0449	*YHI9* – Uncharacterized isomerase Yhi9
YMR088C	1.26	0.0347	*VBA1* – Vacuolar basic amino acid transporter 1
YIL056W	1.26	0.0282	*VHR1* – Transcription factor Vhr1
YGR245C	1.26	0.0346	*SDA1* – Protein Sda1
YCR017C	1.26	0.0076	*CWH43* – Protein Cwh43
YJL163C	1.26	0.0177	*YJQ3* – Uncharacterized membrane protein Yjl163c
YNL237W	1.26	0.0126	*YTP1* – Protein Ytp1
YML075C	1.26	0.0401	*HMDH1* – 3-hydroxy-3-methylglutaryl-coenzyme A reductase 1
YCL030C	1.25	0.0389	*HIS2* – Histidine biosynthesis trifunctional protein
YMR031C	1.25	0.0157	*EIS1* – Eisosome protein 1
YJL078C	1.25	0.0270	*PRY3* – Cell wall protein Pry3
YDL138W	1.25	0.0075	*RGT2* – High glucose sensor Rgt2
YJR106W	1.25	0.0490	*ECM27* – Protein Ecm27
YBR074W	1.25	0.0278	*PFF1* – Vacuolar membrane protease
YNL190W	1.25	0.0177	-
YOR303W	1.24	0.0058	*CARA* – Carbamoyl phosphate synthase arginine-specific small chain

Gene ID	Fold change	Adjusted p-value	Gene standard name and function
YGL006W	1.24	0.0341	*ATC2* – Calcium-transporting ATPase 2
YLR299W	1.24	0.0203	*ECM38* – Glutathione hydrolase proenzyme
YOR002W	1.24	0.0124	*ALG6* – Dolichyl pyrophosphate Man9GlcNAc2 alpha-1,3-glucosyltransferase
YDR536W	1.24	0.0175	*STL1* – Sugar transporter Stl1
YGR260W	1.24	0.0102	*TNA1* – High-affinity nicotinic acid transporter
YLL055W	1.24	0.0245	*YCT1* – High affinity cysteine transporter
YCR032W	1.24	0.0486	*BPH1* – Beige protein homolog 1
YML130C	1.23	0.0199	*ERO1* – Endoplasmic oxidoreductin-1
YJR109C	1.23	0.0449	*CARB* – Carbamoyl phosphate synthase arginine-specific large chain
YNL311C	1.23	0.0432	*SKP2* – F-box protein Skp2
YDR128W	1.23	0.0305	*WDR59* – Maintenance of telomere capping protein 5
YNL142W	1.23	0.0281	*MEP2* – Ammonium transporter Mep2
YDL237W	1.23	0.0305	*AIM6* – Altered inheritance of mitochondria protein 6
YCR098C	1.23	0.0351	*GIT1* – Glycerophosphoinositol transporter 1
YDL054C	1.22	0.0177	*MCH1* – Probable transporter Mch1
YMR297W	1.22	0.0184	*CBPY* – Carboxypeptidase Y
YDR422C	1.22	0.0305	*SIP1* – SNF1 protein kinase subunit beta-1

Gene ID	Fold change	Adjusted p-value	Gene standard name and function
YPL265W	1.22	0.0068	*DIP5* – Dicarboxylic amino acid permease
YMR243C	1.22	0.0199	*ZRC1* – Vacuolar zinc transporter Zrc1
novel.13	1.22	0.0218	*TY1AB* – Transposon TyH3 Gag-Pol polyprotein
YJL045W	1.22	0.0463	*SDHX* – Succinate dehydrogenase [ubiquinone] flavoprotein subunit 2, mitochondrial
YNL280C	1.22	0.0278	*ERG24* – Delta(14)-sterol reductase Erg24
YOR256C	1.21	0.0389	*TRE2* – Putative zinc metalloprotease Tre2
YML006C	1.21	0.0315	*GIS4* – Protein Gis4
YLR207W	1.21	0.0277	*HRD3* – ERAD-associated E3 ubiquitin-protein ligase component Hrd3
YDR492W	1.21	0.0341	*IZH1* – ADIPOR-like receptor Izh1
YDR483W	1.21	0.0245	*KRE2* – Glycolipid 2-alpha-mannosyltransferase
YBR286W	1.21	0.0109	*APE3* – Aminopeptidase Y
YNL268W	1.21	0.0204	*LYP1* – Lysine-specific permease
YDL128W	1.20	0.0490	*VCX1* – Vacuolar calcium ion transporter
YPR083W	1.20	0.0313	*MDM36* – Mitochondrial distribution and morphology protein 36
YGR060W	1.18	0.0306	*ERG25* – C-4 methylsterol oxidase Erg25
YFL016C	0.86	0.0389	*MDJ1* – DnaJ homolog 1, mitochondrial

Gene ID	Fold change	Adjusted p-value	Gene standard name and function
YOR187W	0.85	0.0278	*EFTU* – Elongation factor Tu, mitochondrial
YJR101W	0.85	0.0490	*RT26* – Small ribosomal subunit protein mS42
YKL195W	0.85	0.0203	*MIA40* – Mitochondrial intermembrane space import and assembly protein 40
YDR316W	0.84	0.0278	*OMS1* – Methyltransferase OMS1, mitochondrial
YMR024W	0.84	0.0490	*RM03* – Large ribosomal subunit protein mL44
YLR069C	0.84	0.0346	*EFGM* – Elongation factor G, mitochondrial
YHR174W	0.84	0.0401	*ENO1* – Enolase 1
YOR327C	0.83	0.0346	*SNC2* – Synaptobrevin homolog 2
YKL216W	0.83	0.0422	*PYRD* – Dihydroorotate dehydrogenase (fumarate)
YPR101W	0.83	0.0297	*SN309* – Pre-mRNA-splicing factor Snt309
YLR314C	0.83	0.0203	*CDC3* – Cell division control protein 3
YGL068W	0.82	0.0285	*MNP1* – Large ribosomal subunit protein bL12m
YNL056W	0.82	0.0389	*OCA2* – Tyrosine-protein phosphatase-like protein Oca2
YCL034W	0.82	0.0282	*LSB5* – LAS seventeen-binding protein 5
YIL070C	0.82	0.0199	*MAM33* – Mitochondrial acidic protein Mam33
YGR189C	0.82	0.0278	*CRH1* – Probable glycosidase Crh1
YOR195W	0.81	0.0207	*SLK19* – Kinetochore protein Slk19

Gene ID	Fold change	Adjusted p-value	Gene standard name and function
YNL058C	0.81	0.0087	*YNF8* – Vacuolar membrane protein Ynl058c
YJR048W	0.81	0.0157	*CYC1* – Cytochrome c isoform 1
YHR137W	0.80	0.0390	*ARO9* – Aromatic amino acid aminotransferase 2
YHR209W	0.80	0.0122	*CRG1* – Probable S-adenosylmethionine-dependent methyltransferase Crg1
YJR076C	0.80	0.0138	*CDC11* – Cell division control protein 11
YLR168C	0.80	0.0058	*UPS2* – Protein UPS2, mitochondrial
YKL096W	0.80	0.0157	*CWP1* – Cell wall protein Cwp1
YOL012C	0.80	0.0389	*H2AZ* – Histone H2A.Z
YPR158W	0.80	0.0149	*CUR1* – Curing of [URE3] protein 1
YLR151C	0.79	0.0115	*PCD1* – Peroxisomal coenzyme A diphosphatase 1, peroxisomal
YBR010W	0.79	0.0162	*H3* – Histone H3
YFL045C	0.79	0.0389	*PMM* – Phosphomannomutase
YML102W	0.79	0.0184	*CAF1B* – Chromatin assembly factor 1 subunit B
YHR059W	0.79	0.0281	*FYV4* – Small ribosomal subunit protein mS41
YDR315C	0.79	0.0277	*IPK1* – Inositol-pentakisphosphate 2-kinase
YFL037W	0.78	0.0218	*TBB* – Tubulin beta chain
YDL157C	0.77	0.0305	*YD157* – Uncharacterized protein YDL157C, mitochondrial
YFR001W	0.77	0.0112	*LOC1* – 60S ribosomal subunit assembly/export protein Loc1

Gene ID	Fold change	Adjusted p-value	Gene standard name and function
YPL255W	0.77	0.0430	*BBP1* – Spindle pole component Bbp1
YGR040W	0.76	0.0297	*KSS1* – Mitogen-activated protein kinase Kss1
YIL004C	0.76	0.0087	*BET1* – Protein transport protein Bet1
YDL101C	0.76	0.0012	*DUN1* – DNA damage response protein kinase Dun1
YER030W	0.76	0.0389	*CHZ1* – Histone H2A.Z-specific chaperone Chz1
YDR013W	0.76	0.0245	*PSF1* – DNA replication complex GINS protein Psf1
YJL153C	0.76	0.0347	*INO1* – Inositol-3-phosphate synthase
YOR346W	0.76	0.0306	*REV1* – DNA repair protein Rev1
YNL090W	0.75	0.0067	*RHO2* – GTP-binding protein Rho2
YLR200W	0.75	0.0158	*PFD6* – Prefoldin subunit 6
YNL126W	0.75	0.0305	*SPC98* – Spindle pole body component Spc98
YOR025W	0.74	0.0075	*HST3* – NAD-dependent histone deacetylase Hst3
YOR373W	0.74	0.0188	*NUD1* – Protein Nud1
YLR353W	0.74	0.0109	*BUD8* – Bud site selection protein 8
YBR088C	0.74	0.0203	*PCNA* – Proliferating cell nuclear antigen
novel.376	0.74	0.0223	*COX17* – Cytochrome c oxidase copper chaperone
YJR107C_A	0.73	0.0326	*YJ107* – Uncharacterized protein YJR107C-A
YCR003W	0.73	0.0199	*RM32* – Large ribosomal subunit protein bL32m

Gene ID	Fold change	Adjusted p-value	Gene standard name and function
YOR247W	0.72	0.0067	*YO248* – Putative uncharacterized protein Yor248w
YDR451C	0.72	0.0263	*YHP1* – Homeobox protein Yhp1
novel.458	0.71	0.0083	*STF1* – F(1)-ATPase inhibitor STF1, mitochondrial
YMR003W	0.71	0.0463	*AIM34* – Altered inheritance of mitochondria protein 34
YDL219W	0.71	0.0305	*DTD* – D-aminoacyl-tRNA deacylase
YDR224C	0.70	0.0075	*H2B1* – Histone H2B.1
YBR009C	0.69	0.0029	*H4* – Histone H4
YBL003C	0.68	0.0071	*H2A2* – Histone H2A.2
YGR096W	0.67	0.0134	*TPC1* – Mitochondrial thiamine pyrophosphate carrier 1
novel.439	0.66	0.0395	-
novel.291	0.65	0.0357	*RL6A* – Large ribosomal subunit protein eL6A
YPR078C	0.63	0.0091	*YP078* – Uncharacterized protein Ypr078c
YNL030W	0.63	0.0007	*H4* – Histone H4
YDR222W	0.63	0.0218	*YDR22* – SVF1-like protein Ydr222w
YDR209C	0.59	0.0199	*YD209* – Uncharacterized protein Ydr209c
novel.384	0.53	0.0389	*RUXF* – Small nuclear ribonucleoprotein F
novel.404	0.49	0.0414	-
YFR035C	0.46	0.0346	*YFK5* – Uncharacterized protein Yfr035c
novel.225	0.35	0.0282	-

Gene ID	Fold change	Adjusted p-value	Gene standard name and function
YBL044W	0.02	0.0188	*YBE4* – Uncharacterized protein Ybl044w

Table continued on next page.

3) *WT/rif2Δ* vs *WT/WT*

**Gene ID**	**Fold change**	**Adjusted p-value**	**Gene standard name and function**
novel.1	8.00	1.92E-11	*REP1* – Partitioning protein Rep1
novel.4	4.55	2.32E-07	*FLP* – Site-specific recombinase Flp
YGL028C	2.03	1.65E-11	*SCW11* – Probable family 17 glucosidase Scw11
YJL116C	1.82	1.44E-06	*NCA3* – Beta-glucosidase-like protein NCA3, mitochondrial
YER124C	1.82	3.02E-10	*DSE1* – Protein Dse1
YHR143W	1.80	1.18E-08	*DSE2* – Protein Dse2
YHR096C	1.79	3.08E-12	*HXT5* – Probable glucose transporter Hxt5
YDL222C	1.69	2.82E-14	*FMP45* – SUR7 family protein Fmp45
YBR296C	1.68	0.0004	*PHO89* – Phosphate permease Pho89
YER153C	1.67	0.0477	*PT122* – Protein PET122, mitochondrial
YJL078C	1.65	4.73E-06	*PRY3* – Cell wall protein Pry3
YML123C	1.65	8.80E-09	*PHO84* – Inorganic phosphate transporter Pho84
novel.417	1.63	1.26E-09	*PPA5* – Repressible acid phosphatase
YDL223C	1.63	1.44E-06	*HBT1* – Protein Hbt1
YDL210W	1.62	5.35E-09	*UGA4* – GABA-specific permease
YNR067C	1.60	1.00E-06	*ENG1* – Glucan endo-1,3-beta-D-glucosidase 1
YLR286C	1.59	1.95E-07	*CHIT* – Endochitinase
YNL160W	1.58	3.11E-13	*YGP1* – Protein Ygp1

**Gene ID**	**Fold change**	**Adjusted p-value**	**Gene standard name and function**
YNL327W	1.55	3.08E-06	*EGT2* – Protein Egt2
YPL036W	1.54	0.0047	*PMA2* – Plasma membrane ATPase 2
YDR536W	1.51	3.73E-08	*STL1* – Sugar transporter Stl1
YGR079W	1.50	0.0152	*YG2G* – Putative uncharacterized protein Ygr079w
YNL142W	1.47	6.26E-05	*MEP2* – Ammonium transporter Mep2
YML116W	1.47	0.0027	*ATR1* – Aminotriazole resistance protein
YGL008C	1.47	0.0057	*PMA1* – Plasma membrane ATPase 1
YPL223C	1.46	0.0365	*GRE1* – Protein Gre1
YCR061W	1.46	0.0149	*YCV1* – Uncharacterized membrane protein Ycr061w
YML075C	1.46	0.0005	*HMDH1* – 3-hydroxy-3-methylglutaryl-coenzyme A reductase 1
YBR054W	1.45	1.21E-07	*YRO2* – Protein Yro2
YJL079C	1.45	0.0072	*PRY1* – Protein Pry1
YOL119C	1.45	4.40E-06	*MCH4* – Probable transporter Mch4
YOL155C	1.45	0.0025	*HPF1* – Haze protective factor 1
YNL066W	1.43	0.0045	*SUN4* – Probable secreted beta-glucosidase Sun4
YMR034C	1.43	4.84E-06	*RCH1* – Solute carrier Rch1
YGR260W	1.43	6.63E-06	*TNA1* – High-affinity nicotinic acid transporter
YJR025C	1.42	0.0084	*3HAO* – 3-hydroxyanthranilate 3,4-dioxygenase

Gene ID	Fold change	Adjusted p-value	Gene standard name and function
YPL265W	1.42	1.95E-07	*DIP5* – Dicarboxylic amino acid permease
YPL092W	1.42	8.17E-05	*SSU1* – Sulfite efflux pump Ssu1
YLR312C	1.41	0.0002	*ATG39* – Autophagy-related protein 39
YCR098C	1.40	0.0003	*GIT1* – Glycerophosphoinositol transporter 1
YHL016C	1.40	0.0082	*DUR3* – Urea active transporter
YGL055W	1.39	1.61E-05	*ACO1* – Acyl-CoA desaturase 1
YKL034W	1.38	0.0040	*TUL1* – Transmembrane E3 ubiquitin-protein ligase 1
YCR068W	1.37	5.30E-05	*ATG15* – Putative lipase Atg15
YOL101C	1.37	0.0040	*IZH4* – ADIPOR-like receptor Izh4
YKL187C	1.37	0.0063	*YKS7* – Uncharacterized protein Ykl187c
YLL055W	1.36	0.0001	*YCT1* – High affinity cysteine transporter
YDR205W	1.36	0.0031	*MSC2* – Probable zinc transporter Msc2
YOR348C	1.36	0.0016	*PUT4* – Proline-specific permease
YNL237W	1.35	0.0012	*YTP1* – Protein Ytp1
YKL198C	1.35	0.0058	*PTK1* – Serine/threonine-protein kinase PTK1/STK1
YCR017C	1.35	0.0004	*CWH43* – Protein Cwh43
YJL042W	1.35	0.0332	*MHP1* – MAP-homologous protein 1
YCR021C	1.35	0.0019	*HSP30* – 30 kDa heat shock protein

Gene ID	Fold change	Adjusted p-value	Gene standard name and function
YDL180W	1.34	0.0047	*YD180* – Uncharacterized membrane protein Ydl180w
YMR088C	1.34	0.0147	*VBA1* – Vacuolar basic amino acid transporter 1
YGR197C	1.34	0.0045	*SNG1* – Nitrosoguanidine resistance protein Sng1
YJL172W	1.33	0.0166	*CBPS* – Carboxypeptidase S
YJR078W	1.33	0.0470	*I23O* – Indoleamine 2,3-dioxygenase
YMR266W	1.33	0.0232	*RSN1* – Uncharacterized protein Rsn1
YBR235W	1.32	0.0115	*VHC1* – Vacuolar cation-chloride cotransporter 1
YJR152W	1.32	0.0059	*DAL5* – Allantoate permease
novel.134	1.32	0.0062	*TPO3* – Polyamine transporter 3
novel.9	1.32	0.0332	*YP202* – Putative uncharacterized protein Ypr202w
YER052C	1.32	0.0035	*AK* – Aspartokinase
YDR033W	1.31	0.0047	*MRH1* – Protein Mrh1
YGL186C	1.31	0.0032	*TPN1* – Vitamin B6 transporter Tpn1
YLR207W	1.31	0.0023	*HRD3* – ERAD-associated E3 ubiquitin-protein ligase component Hrd3
YNL195C	1.31	0.0176	*YNT5* – Uncharacterized protein Ynl195c
YHR029C	1.31	0.0488	*YHI9* – Uncharacterized isomerase Yhi9
YLR273C	1.31	0.0468	*PIG1* – Serine/threonine-protein phosphatase 1 regulatory subunit Pig1

Gene ID	Fold change	Adjusted p-value	Gene standard name and function
YDR135C	1.30	0.0214	*YCFI* – Metal resistance protein Ycf1
YJR116W	1.30	0.0165	*TDA4* – Topoisomerase I damage affected protein 4
YLR299W	1.30	0.0054	*ECM38* – Glutathione hydrolase proenzyme
YIL030C	1.30	0.0161	*DOA10* – ERAD-associated E3 ubiquitin-protein ligase Doa10
YBR229C	1.30	0.0272	*GLU2A* – Glucosidase 2 subunit alpha
YGL027C	1.30	0.0302	*CWH41* – Mannosyl-oligosaccharide glucosidase
YNR002C	1.29	0.0058	*ATO2* – Ammonia transport outward protein 2
YKL185W	1.29	0.0135	*ASH1* – Transcriptional regulatory protein Ash1
YIL090W	1.29	0.0121	*ICE2* – Protein Ice2
YDR284C	1.29	0.0057	*DPP1* – Diacylglycerol pyrophosphate phosphatase 1
YGL117W	1.29	0.0332	*YGL7* – Uncharacterized protein Ygl117w
YLR152C	1.28	0.0100	*YL152* – Uncharacterized transporter Ylr152c
YPR026W	1.28	0.0326	*ATH1* – Periplasmic acid trehalase Atc1
YGL146C	1.28	0.0448	*RRT6* – Regulator of rDNA transcription protein 6
YDL237W	1.28	0.0139	*AIM6* – Altered inheritance of mitochondria protein 6
YJL045W	1.28	0.0273	*SDHX* – Succinate dehydrogenase [ubiquinone] flavoprotein subunit 2, mitochondrial

Gene ID	Fold change	Adjusted p-value	Gene standard name and function
YDR090C	1.28	0.0074	*YD090* – Uncharacterized membrane protein Ydr090c
YKL217W	1.28	0.0031	*JEN1* – Carboxylic acid transporter protein homolog
YOR002W	1.28	0.0121	*ALG6* – Dolichyl pyrophosphate Man9GlcNAc2 alpha-1,3-glucosyltransferase
YOL058W	1.28	0.0187	*ASSY* – Argininosuccinate synthase
YJL062W	1.27	0.0161	*GPI7* – GPI ethanolamine phosphate transferase 2
YDL093W	1.27	0.0141	*PMT5* – Dolichyl-phosphate-mannose--protein mannosyltransferase 5
YDR492W	1.27	0.0060	*IZH1* – ADIPOR-like receptor Izh1
YOR303W	1.27	0.0025	*CARA* – Carbamoyl phosphate synthase arginine-specific small chain
YMR221C	1.27	0.0199	*FMP42* – Protein Fmp42
YCR010C	1.26	0.0081	*ADY2* – Accumulation of dyads protein 2
YGL006W	1.26	0.0439	*ATC2* – Calcium-transporting ATPase 2
YDR307W	1.26	0.0223	*PMT7* – Probable dolichyl-phosphate-mannose--protein mannosyltransferase 7
YPL274W	1.26	0.0419	*SAM3* – S-adenosylmethionine permease Sam3
YNL194C	1.26	0.0174	*YNT4* – Uncharacterized plasma membrane protein Ynl194c
YMR243C	1.26	0.0100	*ZRC1* – Vacuolar zinc transporter Zrc1
YJL163C	1.26	0.0159	*YJQ3* – Uncharacterized membrane protein Yjl163c

Gene ID	Fold change	Adjusted p-value	Gene standard name and function
YDL054C	1.26	0.0180	*MCH1* – Probable transporter Mch1
YBR241C	1.26	0.0067	*YB91* – Probable metabolite transport protein Ybr241c
YPL026C	1.26	0.0273	*SKS1* – Serine/threonine-protein kinase Sks1
YOL013C	1.25	0.0127	*HRD1* – ERAD-associated E3 ubiquitin-protein ligase Hrd1
YDR483W	1.25	0.0121	*KRE2* – Glycolipid 2-alpha-mannosyltransferase
YJR013W	1.25	0.0433	*GPI14* – GPI mannosyltransferase 1
YMR297W	1.25	0.0131	*CBPY* – Carboxypeptidase Y
YBR072W	1.25	0.0327	*HSP26* – Heat shock protein 26
YGR060W	1.25	0.0062	*ERG25* – C-4 methylsterol oxidase Erg25
YKL004W	1.25	0.0408	*AUR1* – Inositol phosphorylceramide synthase catalytic subunit Aur1
YBR069C	1.25	0.0176	*TAT1* – Valine/tyrosine/tryptophan amino-acid permease 1
YHR039C	1.25	0.0177	*MSC7* – Putative aldehyde dehydrogenase-like protein Yhr039c
YGR121C	1.25	0.0332	*MEP1* – Ammonium transporter Mep1
YML128C	1.25	0.0122	*MSC1* – Meiotic sister chromatid recombination protein 1
YPR083W	1.25	0.0161	*MDM36* – Mitochondrial distribution and morphology protein 36
YHR202W	1.25	0.0141	*YH02* – Uncharacterized protein Yhr202w

Gene ID	Fold change	Adjusted p-value	Gene standard name and function
YNL268W	1.24	0.0139	*LYP1* – Lysine-specific permease
YER056C	1.24	0.0301	*FCY2* – Purine-cytosine permease Fcy2
YKR067W	1.24	0.0214	*GPT2* – Glycerol-3-phosphate O-acyltransferase 2
YKL140W	1.24	0.0165	*TGL1* – Sterol esterase Tgl1
YGL022W	1.24	0.0479	*STT3* – Dolichyl-diphosphooligosaccharide--protein glycosyltransferase subunit Stt3
YCL025C	1.24	0.0232	*AGP1* – General amino acid permease Agp1
YAL062W	1.24	0.0259	*DHE5* – NADP-specific glutamate dehydrogenase 2
YKL207W	1.24	0.0334	*EMC3* – ER membrane protein complex subunit 3
YLR056W	1.24	0.0205	*ERG3* – Delta(7)-sterol 5(6)-desaturase Erg3
YDR233C	1.23	0.0131	*RTN1* – Reticulon-like protein 1
YMR015C	1.23	0.0098	*ERG5* – C-22 sterol desaturase Erg5
YNL280C	1.23	0.0333	*ERG24* – Delta(14)-sterol reductase Erg24
novel.514	1.23	0.0292	*G3P1* – Glyceraldehyde-3-phosphate dehydrogenase 1
YBR110W	1.23	0.0314	*ALG1* – Chitobiosyldiphosphodolichol beta-mannosyltransferase
YFL041W	1.22	0.0327	*FET5* – Iron transport multicopper oxidase Fet5
YBR067C	1.22	0.0135	*TIP1* – Temperature shock-inducible protein 1

Gene ID	Fold change	Adjusted p-value	Gene standard name and function
YHR188C	1.22	0.0317	*GPI16* – GPI transamidase component Gpi16
YIL088C	1.22	0.0414	*AVT7* – Vacuolar amino acid transporter 7
YGR284C	1.22	0.0433	*ERV29* – ER-derived vesicles protein Erv29
YGL077C	1.22	0.0333	*HNM1* – Choline transporter
YDL204W	1.22	0.0179	*RTN2* – Reticulon-like protein 2
YOR316C	1.22	0.0259	*COT1* – Vacuolar zinc transporter Cot1
novel.358	1.21	0.0419	*ATR* – Serine/threonine-protein kinase Mec1
YHR132C	1.21	0.0317	*ECM14* – Inactive metallocarboxypeptidase Ecm14
YFL016C	0.82	0.0152	*MDJ1* – DnaJ homolog 1, mitochondrial
YOR187W	0.82	0.0098	*EFTU* – Elongation factor Tu, mitochondrial
YNL067W	0.81	0.0456	*RL9B* – Large ribosomal subunit protein uL6B
YPR047W	0.81	0.0397	*SYFM* – Phenylalanine--tRNA ligase, mitochondrial
YGR161C	0.81	0.0289	*RTS3* – Protein phosphatase type 2A regulatory subunit Rts3
YER077C	0.81	0.0412	*MRX1* – MIOREX complex component 1
YDR381W	0.80	0.0343	*YRA1* – RNA annealing protein Yra1
YOL096C	0.80	0.0423	*COQ3* – Ubiquinone biosynthesis O-methyltransferase, mitochondrial
YMR193W	0.79	0.0175	*RM24* – Large ribosomal subunit protein bL28m

Gene ID	Fold change	Adjusted p-value	Gene standard name and function
YMR024W	0.79	0.0124	*RM03* – Large ribosomal subunit protein mL44
YPR158W	0.78	0.0345	*CUR1* – Curing of [URE3] protein 1
YJR094W_A	0.78	0.0400	*RL43A* – Large ribosomal subunit protein eL43A
YKL216W	0.78	0.0031	*PYRD* – Dihydroorotate dehydrogenase (fumarate)
YLR069C	0.78	0.0031	*EFGM* – Elongation factor G, mitochondrial
YHR209W	0.77	0.0165	*CRG1* – Probable S-adenosylmethionine-dependent methyltransferase Crg1
YPR101W	0.77	0.0040	*SN309* – Pre-mRNA-splicing factor Snt309
YBL078C	0.76	0.0098	*ATG8* – Autophagy-related protein 8
YOR195W	0.75	0.0019	*SLK19* – Kinetochore protein Slk19
YOR131C	0.73	0.0056	*YOR31* – Uncharacterized hydrolase Yor131c
YBR291C	0.72	0.0294	*TXTP* – Tricarboxylate transport protein
YDL165W	0.71	0.0027	*NOT2* – General negative regulator of transcription subunit 2
YIR042C	0.68	0.0012	*YIW2* – Uncharacterized protein Yir042c
YLR160C	0.62	5.13E-09	*ASP21* – L-asparaginase 2-1
YLR453C	0.59	0.0056	*RIF2* – Protein Rif2
YLR161W	0.46	0.0030	*YL156* – Putative uncharacterized protein Ylr156w

II. Heatmap of significantly differentially expressed genes (adjusted p ≤ 0.05) across the three pairwise comparisons

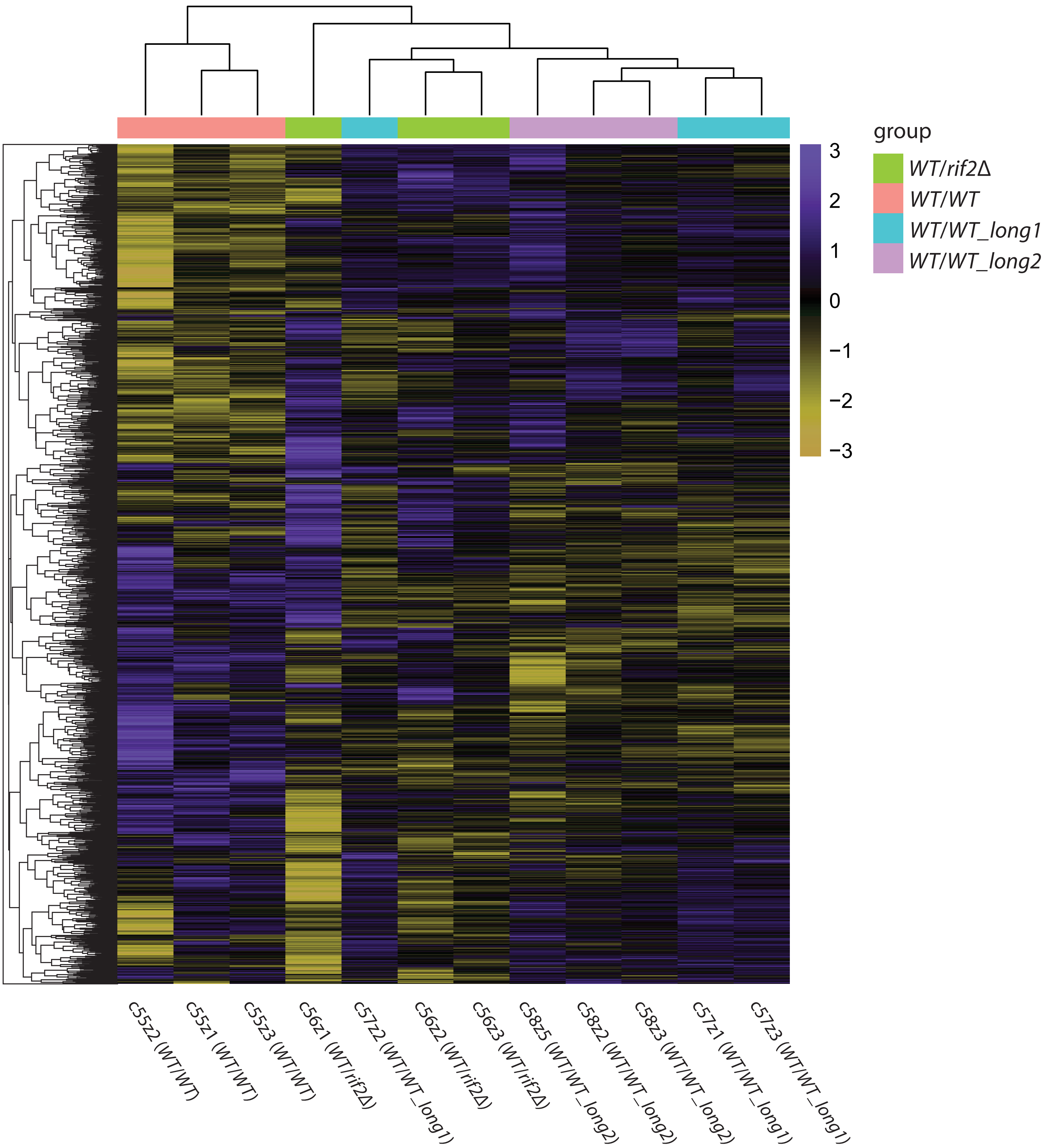

III. Venn diagram illustrating the overlap between significantly differentially expressed genes (adjusted p ≤ 0.05) across the three pairwise comparisons

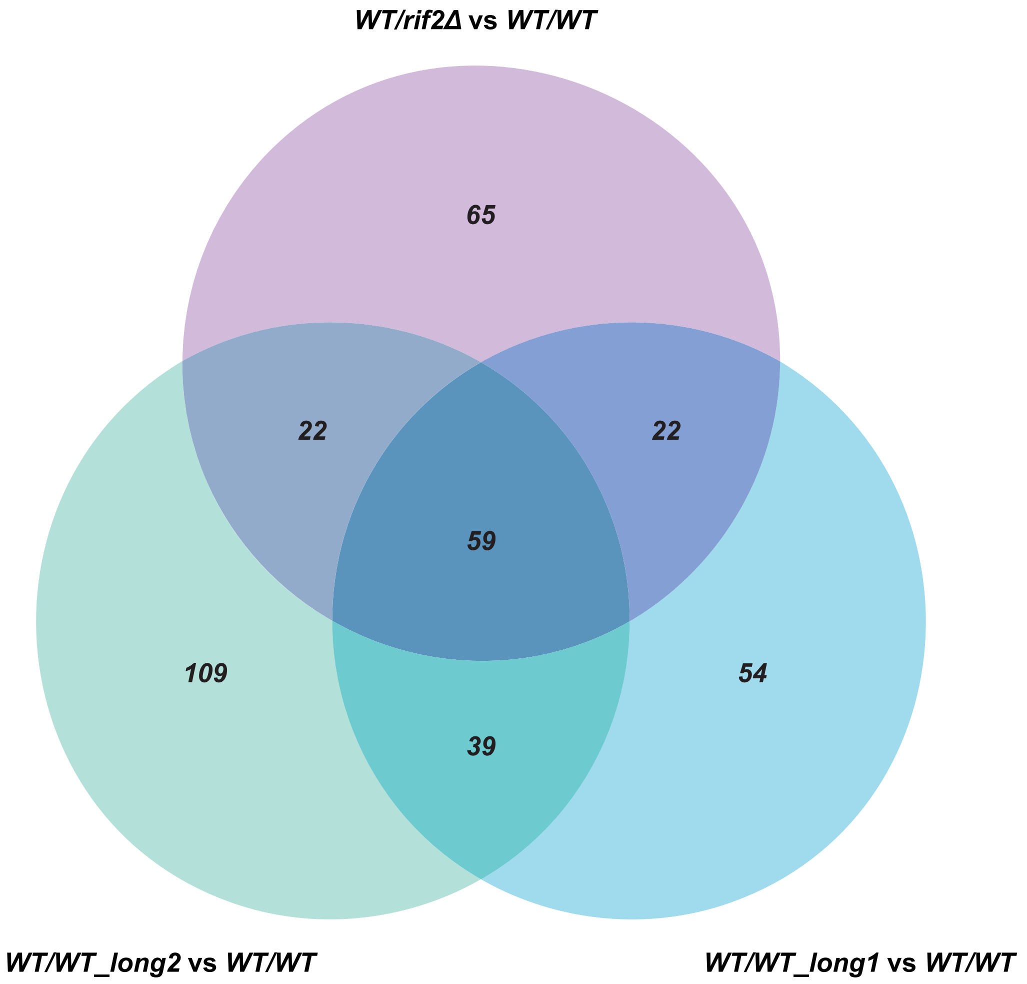

IV. Gene ontology analysis of the 59 DEGs shared across the three pairwise comparisons

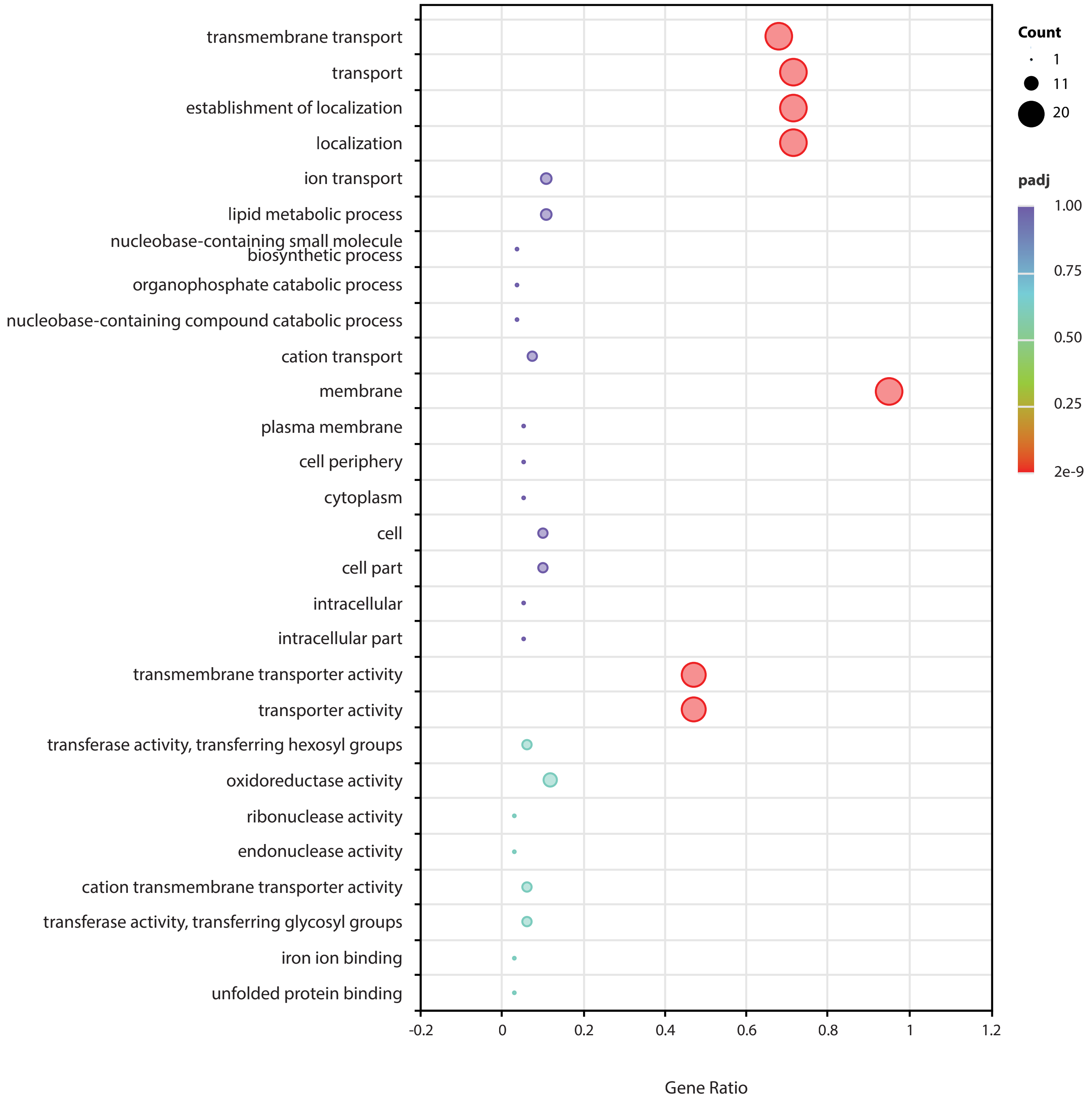

V. Genomic position and Rap1-binding status of the 59 DEGs

Gene ID	Gene standard name and function	Chromosome number	Distance from telomere (kb)	Binds Rap1?
YML128C	*MSC1* – Meiotic sister chromatid recombination protein	XIII	15	No
YBR296C	*PHO89* – Phosphate permease Pho89	II	15	Yes
YDR536W	*STL1* – Sugar transporter Stl1	IV	23	No
YML123C	*PHO84* – Inorganic phosphate transporter Pho84	XIII	24	Yes
YKL216W	*URA1* – Dihydroorotate dehydrogenase (fumarate)	XI	25	No
YDL237W	*AIM6* – Altered inheritance of mitochondria protein 6	IV	30	No
YLL055W	*YCT1* – High affinity cysteine transporter	XII	30	Yes
YML116W	*ATR1* – Aminotriazole resistance protein	XIII	38	No
YPL265W	*DIP5* – Dicarboxylic amino acid permease	XVI	41	No
YDL223C	*HBT1* – Protein Hbt1	IV	57	No
YHR202W	*SMN1* – Putative AMP hydrolase	VIII	60	No
YHL016C	*DUR3* – Urea active transporter	VIII	72	Yes
YGR260W	*TNA1* – High-affinity nicotinic acid transporter	VII	78	Yes
YCR068W	*ATG15* – Putative lipase Atg15	III	79	Yes
YDL210W	*UGA4* – GABA-specific permease	IV	84	No
YKL187C	*FAT3* – Fatty acid transporter	XI	89	No
YCR061W	*YCV1* – Uncharacterized membrane protein Ycr061w	III	91	No

Gene ID	Gene standard name and function	Chromosome number	Distance from telomere (kb)	Binds Rap1?
YDL204W	*RTN2* – Reticulon-like protein	IV	94	Yes
YOL119C	*MCH4* – Probable transporter Mch4	XV	95	Yes
YDR492W	*IZH1* – ADIPOR-like receptor Izh1	IV	97	No
YJL172W	*CBPS* – Carboxypeptidase S	X	98	No
YFL016C	*MDJ1* – DnaJ homolog 1, mitochondrial	VI	104	Yes
YPR158W	*CUR1* – Curing of [URE3] protein	XVI	105	No
YDR483W	*KRE2* – Glycolipid 2-alpha-mannosyltransferase	IV	110	No
novel.134	*TPO3* – Polyamine transporter	XVI	111	?
YJL163C	*YJQ3* – Uncharacterized membrane protein Yjl163c	X	112	No
YML075C	*HMDH1* – 3-hydroxy-3-methylglutaryl-coenzyme A reductase	XIII	115	No
YMR266W	*RSN1* – Uncharacterized protein Rsn1	XIII	126	Yes
YBR235W	*VHC1* – Vacuolar cation-chloride cotransporter	II	127	No
YNL268W	*LYP1* – Lysine-specific permease	XIV	138	Yes
YCR017C	*CWH43* – Protein Cwh43	III	144	No
YGL186C	*TPN1* – Vitamin B6 transporter Tpn1	VII	151	No
YIL030C	*DOA10* – ERAD-associated E3 ubiquitin-protein ligase Doa10	IX	154	No
YCR021C	*HSP30* – 30 kDa heat shock protein	III	160	No
YJL116C	*NCA3* – Beta-glucosidase-like protein NCA3, mitochondrial	X	194	Yes
YNL237W	*YTP1* – Protein Ytp1	XIV	205	No

Gene ID	Gene standard name and function	Chromosome number	Distance from telomere (kb)	Binds Rap1?
YHR096C	*HXT5* – Probable glucose transporter Hxt5	VIII	268	Yes
YNL195C	*YNT5* – Uncharacterized protein Ynl195c	XIV	271	No
YLR069C	*EFGM* – Elongation factor G, mitochondrial	XII	271	No
YKL034W	*TUL1* – Transmembrane E3 ubiquitin-protein ligase 1	XI	295	No
YMR024W	*RM03* – Large ribosomal subunit protein mL44	XIII	321	No
YOR002W	*ALG6* – Dolichyl pyrophosphate Man9GlcNAc2 alpha-1,3-glucosyltransferase	XV	329	No
YNL160W	*YGP1* – Protein Ygp1	XIV	336	No
YMR034C	*RCH1* – Solute carrier Rch1	XIII	339	No
YBR054W	*YRO2* – Protein Yro2	II	343	No
YLR299W	*ECM38* – Glutathione hydrolase proenzyme	XII	352	Yes
YJL045W	*SDH9* – Succinate dehydrogenase [ubiquinone] flavoprotein subunit 2, mitochondrial	X	356	Yes
YNL142W	*MEP2* – Ammonium transporter Mep2	XIV	357	Yes
YDL054C	*MCH1* – Probable transporter Mch1	IV	360	Yes
YBR067C	*TIP1* – Temperature shock-inducible protein	II	372	No
YGL055W	*OLE1* – Acyl-CoA desaturase	VII	399	Yes
YOR187W	*EFTU* – Elongation factor Tu, mitochondrial	XV	407	No
YPL036W	*PMA2* – Plasma membrane ATPase	XVI	466	Yes
YGL008C	*PMA1* – Plasma membrane ATPase	VII	479	Yes

**Gene ID**	**Gene standard name and function**	**Chromosome number**	**Distance from telomere (kb)**	**Binds Rap1?**
YGR060W	*ERG25* – C-4 methylsterol oxidase Erg25	VII	480	Yes
YGL006W	*PMC1* – Calcium-transporting ATPase 2	VII	486	No
YLR207W	*HRD3* – ERAD-associated E3 ubiquitin-protein ligase component Hrd3	XII	522	No
YDR135C	*YCFI* – Metal resistance protein Ycf1	IV	723	Yes
YDR205W	*MSC2* – Probable zinc transporter Msc2	IV	772	No

VI. Relationship between |log$_2$FC| and distance from the nearest telomere (kb) for the 59 DEGs

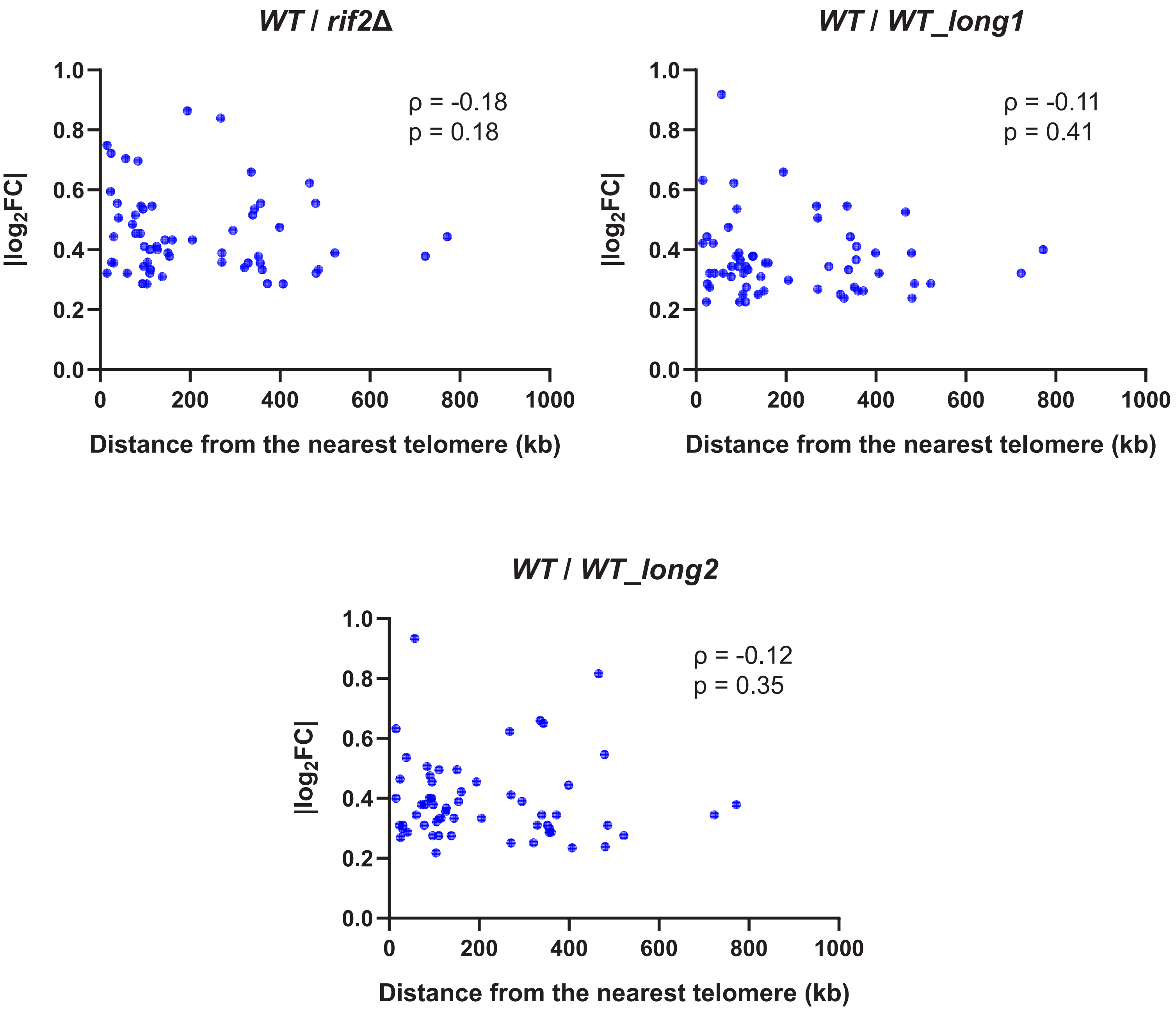

VII. Per-gene changes in |log_2FC| across long-telomere strains for the 59 DEGs

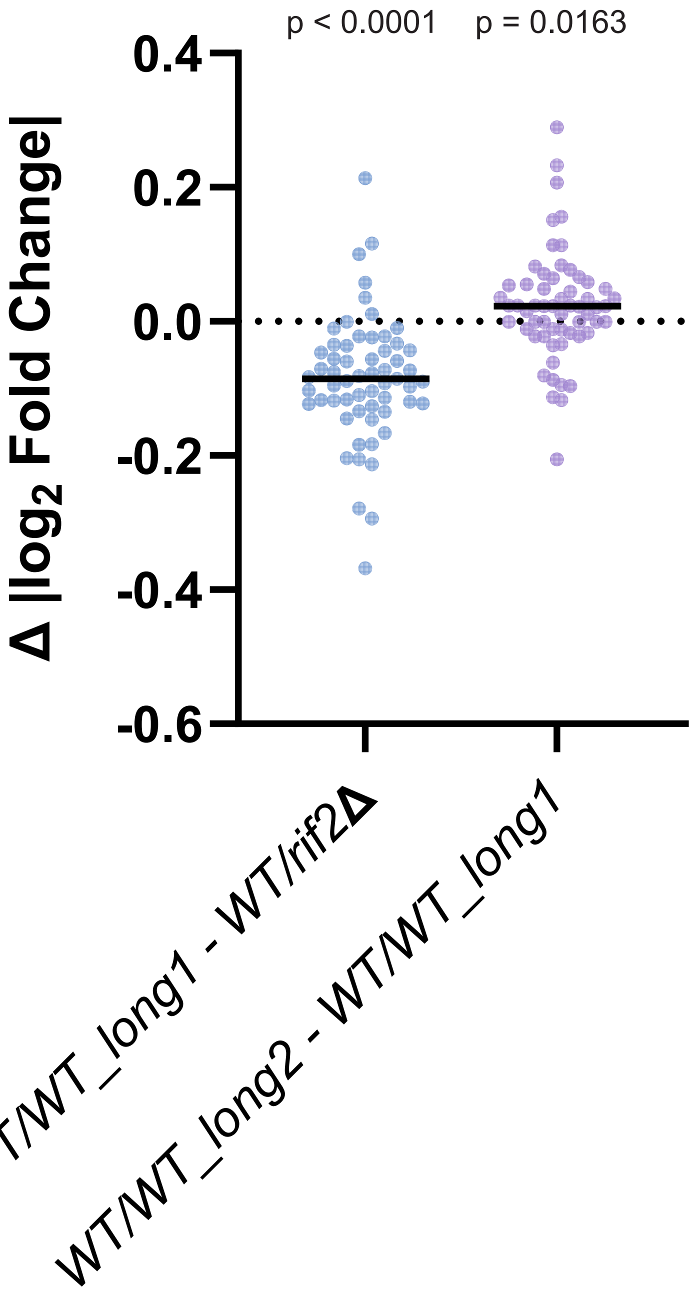

Supplement: Supplementary file 1 [file iyag174_supplementary_data.zip › File_S2_GENETICS-2026-309600.pdf]
