# Supplementary material for: Inherited long telomeres induce a genome-wide transcriptional response in budding yeast
Source: Genetics. 2026 Jul 3;234(2):iyag174. doi: 10.1093/genetics/iyag174 (PMC13643770; doi:10.1093/genetics/iyag174)
Supplement: Supplementary file 1 [file iyag174_supplementary_data.zip › Supplemental_Material_Legends_GENETICS-2026-309600.docx]

**Supplemental Material legends**

**Figure S1**. *Biological replicates clustered consistently by genotype, apart from a single outlier.* Principal component analysis (PCA) of RNA-seq samples based on log₂(FPKM + 1) values. Each point represents one biological replicate, colour- and shape-coded by genotype group. The first two principal components (PC1 and PC2) explain 25.73% and 15.90% of the total variance, respectively. Analyses were also performed including the outlier sample (File S2). Inclusion of this sample would not substantially alter the overall patterns of differential expression or the main conclusions of this study.

**Figure S2**. *Upregulation of transport-related genes represents one of the most consistent responses to telomere elongation.* A scatterplot showing enriched Gene Ontology (GO) terms for differentially expressed genes (DEGs). The x-axis indicates the gene ratio (the fraction of DEGs annotated to a given GO term relative to all DEGs with GO annotations). The y-axis lists GO terms. Dot size reflects the number of DEGs assigned to each term, and dot colour indicates enrichment significance (adjusted p-value).

**Table S1.** *Overlap between the 57 DEGs and genes affected by Rap1 depletion (Kalra et al. 2023).*

**File S1.** *Lists of differentially expressed genes across the three pairwise comparisons, excluding the outlier RNA-seq replicate c56z1 (WT/rif2∆).*

**File S2.** *Analyses including the outlier RNA-seq replicate c56z1 (WT/rif2∆), comprising pairwise comparisons tables, cluster analysis, Venn diagram of overlapping DEGs, Gene Ontology analysis of overlapping DEGs, a table listing overlapping DEGs, analysis of correlation between |log₂FC| and distance from the nearest telomere, and per-gene changes in |log₂FC| across long-telomere strains.*
